# Bryostatin Activates CAR T-Cell Antigen-Non-Specific Killing (CTAK), and CAR-T NK-Like Killing for Pre-B ALL, While Blocking Cytolysis of a Burkitt Lymphoma Cell Line

**DOI:** 10.3389/fimmu.2022.825364

**Published:** 2022-02-09

**Authors:** Lingyan Wang, Yue Zhang, Eden Anderson, Adam Lamble, Rimas J. Orentas

**Affiliations:** ^1^ Ben Town Center for Childhood Cancer Research, Seattle Children’s Research Institute, Seattle, WA, United States; ^2^ Department of Pediatrics, Hematology, Oncology and Bone Marrow Transplant Division, University of Washington School of Medicine, Seattle, WA, United States

**Keywords:** CAR-T cell, CD22, adoptive immunotherapy, antigen density, innate immunity, T cell, cellular cytotoxicity, acute lymphocytic leukemia (B-ALL)

## Abstract

The advent of CAR-T cell therapy has changed the face of clinical care for relapsed and refractory pre-B-acute lymphocytic leukemia (B-ALL) and lymphoma. Although curative responses are reported, long-term cures remain below 50%. Different CAR T-cell leukemia targets appear to have different mechanisms of CAR-T escape. For CD22, therapeutic evasion is linked to down-modulation of the number CD22 proteins expressed on the extracellular aspect of the leukemia cell plasma membrane. Recently, pharmacologic agents known to induce cellular differentiation or epigenetic modification of leukemia have been shown to impact CD22 and CD19 expression levels on B-ALL, and thereby increase sensitivity to CAR-T mediated cytolysis. We explored the impact of epigenetic modifiers and differentiation agents on leukemia cell lines of B cell origin, as well as normal B cells. We confirmed the activity of bryostatin to increase CD22 expression on model cell lines. However, bryostatin does not change CD22 levels on normal B cells. Furthermore, bryostatin inhibited CAR-T mediated cytolysis of the Raji Burkitt lymphoma cell line. Bryostatin increased the cytolysis by CD22 CAR-T for B-ALL cell lines by at least three mechanisms: 1) the previously reported increase in CD22 target cell numbers on the cell surface, 2) the induction of NK ligands, and 3) the induction of ligands that sensitize leukemia cells to activated T cell antigen-non-specific killing. The opposite effect was seen for Burkitt lymphoma, which arises from a more mature B cell lineage. These findings should caution investigators against a universal application of agents shown to increase killing of leukemia target cells by CAR-T in a specific disease class, and highlights that activation of non-CAR-mediated killing by activated T cells may play a significant role in the control of disease. We have termed the killing of leukemia targets, by a set of cell-surface receptors that does not overlap with NK-like killing “CTAK,” CAR-T Cell antigen-non-specific killing.

## Introduction

Adoptive immunotherapy with chimeric antigen receptor (CAR)-mediated T cells has opened a new chapter in the treatment of relapsed and refractory pre-B cell acute lymphocytic leukemia (B-ALL) in pediatric patients as well as for leukemia and lymphomas of B cell lineage in adults (1). Targets include B-cell restricted antigens expressed early in lineage commitment such as CD19 and CD22, later in development such as CD20, and also in more terminal stages of B cell differentiation such as BCMA (2–8). To overcome antigen loss variation, CAR-T targeting multiple antigens have been proposed, including CD19/CD20 and CD19/CD22 Tandem CARs and HIV-Specific DuoCARs which express three binding moieties (9–11). Unlike the escape from CAR-T immunosurveillance by CD19-CAR, which seems to be primarily due to splice variations and thereby the loss of the CAR-binding epitope, CD22-CAR-T evasion is different (12). Leukemic escape from CD22-targeting CAR-T has been demonstrated clinically to be associated with a down-regulation of the number of CD22 molecules expressed on the cell surface (2). In 2019, Ramakrishna et al., demonstrated that inclusion of bryostatin augmented anti-CD22 CAR activity in murine model systems by increasing CD22 antigen expression on the ALL cell lines NALM6 and KOPN8, two model leukemia cell lines, as well as a patient derived xenograft, building on earlier work in chronic lymphocytic leukemia (CLL) (13, 14).

The expression of cell surface glycoproteins, such as CD22, can be regulated at the level of increased mRNA and protein expression, changes in membrane residence, or alterations in recycling of membrane proteins from endocytic vesicles. The use of epigenetic modifiers or differentiation agents has the ability to regulate each of these processes. Until recently, endocytic recycling was regarded as a largely passive process, and that resident proteins were sorted either for degradation or followed bulk membrane flow back to the surface (15). The endocytic process is now known to feature fast recycling through the early endosome, slow recycling through the endocytic recycling compartment, and in some cases retrograde transport to the Golgi apparatus. Degradation is also a carefully regulated sorting process carried out in the endolysosome, which then later fuses to form a mature lysosome (15). In an detailed study, epidermal growth factor receptor (EFGR) was found to internalize the endosome-associated transcriptional regulatory factor RNF11 which translocates to the nucleus where it regulates endoplasmic reticulum export machinery to promote the movement of newly synthesize EGFR through the Golgi to the cell surface (16). The full control of CD22 membrane residence is still under investigation and will likely change depending upon the differentiation state of the B cell.

We show that exposing leukemia cell lines to anti-CD22 CAR-T also changes CD22 surface expression. CAR-T directly and rapidly modulates CD22 surface expression. Surprisingly, the exposure to CD22 CAR-T also modulated CD19, indicating a generalized mechanism of cell surface membrane regulation that can be used to escape CAR-mediated immune surveillance. Thus, rapid modulation is part and parcel of the CAR-T interaction process with transformed B cells, that can potentially be modulated by epigenetic modifiers. Unexpectedly, we also discovered that bryostatin induces changes in immortalized B cell lines that are dependent on the differentiation state (disease origin) of the transformed cell. For pre-B-ALL model cell lines, not only was the number of CD22 molecules on the surface upregulated, two other types of innate immune targeting molecules or activities were induced. The first activity induced can be classified as sensitization to NK-killing, which can be blocked by the presence of the K562 cell lines. Here, we also describe a non-classical innate immune receptor activity that operates similarly to NK-like killing for activated human T cells, but is not blocked by K562. We refrained from the terminology “LAK cell” as this is reserved for a specific type of immune cell driven by high levels of cytokine alone (17). We refer to this second set of receptors as “activated T cell antigen-non-specific” cell ligands, that engage in “CAR T-cell antigen non-specific killing” (CTAK). This activity is induced by the unique properties of CAR-T manufacturing, and is recognized upon bryostatin treatment of ALL. In direct opposition to the effect on ALL lines, we found that bryostatin profoundly inhibits killing of the Raji Burkitt lymphoma cell line, indicating an essential dependence on B cell differentiation for sensitization to CAR-T cell mediated killing.

## Materials and Methods

### Cell Lines and Culture Media

Three CD22 positive leukemia cell lines were used in this study: Raji, NALM6 and REH. The K562 cell line was used as a negative control. For Luciferase-based cytotoxicity assays, Raji-Luc, NALM6-Luc, REH-Luc and K562-Luc were used as target cells. B-LCL cell lines were used for anti-CD22 CART cells rapid expansion protocol (REP). Raji, NALM6, REH, K562, LCL, Raji-Luc and K562-Luc were provided by Dr. Michael Jensen, Seattle Children’s Research Institute. NALM6-Luc and REH-Luc were produced by transducing NALM6 or REH cells with a Luciferase-expressing lentiviral vector (LV), then positive clones were selected and expanded. STR fingerprinting was conducted to verify the identity of cell lines, and each cell line was validated to be *Mycoplasma* free by qPCR. Cell lines were cultured in RPMI 1640 supplemented with 2 mM l-glutamine, 10 mM HEPES (Invitrogen), and 10% heat-inactivated FBS (VWR). Human PBMCs from healthy donors were obtained from Bloodworks Northwest and isolated with SepMate™ PBMC Isolation Tubes and Lymphoprep (Stemcell Technologies). CART and un-transduced control (UTD) cells were cultured in TexMACS™ medium (Miltenyi Biotec) with recombinant IL-2 (premium grade, Miltenyi Biotec). B cells were cultured in B cell culture media (BCM), including RPMI-1640, 10% FCS, 55 mM 2-ME, 1% Pen Strep, 10 mM HEPES, 1 mM Sodium Pyruvate and 1% MEM NEAA, supplemented with recombinant human IL-2 (50 ng/ml, Miltenyi Biotec), IL-4 (10 ng/ml, PeproTech), IL-21 (10 ng/ml, Miltenyi Biotec), and BAFF (10 ng/ml, PeproTech). NK92 were culture in RPMI medium with 10% heat-inactivated FBS (VWR), 1% NEAA, 1% Sodium Pyruvate, 200U/mL IL-2, 2 mM L-glutamine and 25 mM HEPES.

### Primary B Cell Culture and Expansion

Primary B cell expansion was carried out as per Su, K.Y., et al., with the following modifications (18). Six-well plates were pre-seeded overnight with the MS5-based stromal cell line, CD40L-low (MS40L^low^), kindly provided by Dr. Garnett Kelsoe, Duke University, Durham, NC (19) in BCM. B cells were isolated from 3 individual donors using immunomagnetic bead separation (B cell isolation kit, Miltenyi Biotec), cultured in coated six-well plates, 1x10^3^ per well, in BCM for 8 d, and expanded B cells subsequently harvested and cryopreserved in 90% FBS/10%DMSO until use.

### CAR-T Production

CD22 chimeric antigen receptor (CAR) used in this study consists of a single chain fragment variable (ScFv) sequence derived from m971, CD8a hinge and transmembrane domain, 4-1BB(CD137) and CD3- ζ chain signaling domains, as previously described (20). CD22 CAR-encoding lentiviral vector (LV) was produced by transient transfection of the HEK293T/17SF cell line. 2× 10^8^ HEK293T/17SF cells were seeded into 1L flask (Cole Palmer #EW-06019-30) with 200mL FreeStyle293 expression medium (Gibco). The following day, HEK293T/17SF cells were transfected by PEIpro (Polyplus) with plasmids encoding CD22 CAR, gag-pol, rev and VSV-G envelope protein, and sodium butyrate (MiiliporeSigma) was added at 24 h. After 2 days, supernatant was collected and filtered by 0.45uM filter, LV was concentrated by centrifugation at 10,000 xg for 4hr. Pelleted LV was resuspended in serum-free RPMI medium and stored at -80°C. PBMC were activated with TransAct activation reagent in TexMACS medium (Miltenyi Biotec) supplemented with 40 IU/mL IL-2 at density of 1 x10^6^ cells/ml. Activated T cells were transduced with CD22-CAR LV in the presence of 8 µg/mL protamine sulfate on Day 2 in TexMACS medium supplemented with 40 IU/mL IL-2, and volume increased day 3 with IL-2 containing media. On day 4, cultures were harvested and re-seeded in TexMACS with 200 IU/ml IL-2 and expanded until harvest on day 10–13.

### Rapid Expansion Protocol (REP)

Based on protocols established to expand T cell clones, CAR-T or untransduced control T cells (UTD) were co-incubated with irradiated B-LCL (8000 rads) at a 1:7 ratio in complete RPMI supplemented with IL-2 (50 U/ml), IL-7 (5 ng/ml), and IL-15 (0.5 ng/ml). Cells were passaged every 2-3 days and harvested after 10-13 days of expansion (21) (Riddell S and Greenberg P, US Patent 5,827,642). The REP maintains the original phenotype of expanded CAR-T and T cells clones, and CAR-T and UTD remain CD56 negative (**Supplementary Figure S7N**).

### Biochemical Reagents, Antibodies and Recombinant Proteins

5-azacytidine, Vorinostat, Panobinostat, All-Trans Retinoic Acid (ATRA) and Bryostatin 1 (Sigma) were used to treat Raji, NALM6 and REH cell lines for 48 hours, or 24 hours in the case of Bryostatin. Viability, CD19 and CD22 expression levels were assayed at the end of treatment.

Flow cytometry was performed on a Fortessa (BD Biosciences) and data analyzed with FlowJo software (BD Biosciences). Expression levels of CD19 and CD22 on leukemia lines were measured using Quanti-Brite PE beads (BD Bioscience) and PE-labeled anti-CD19 (BioLegend, clone HIB19) and anti-CD22 (BD Bioscience, clone HIB22) antibodies. To determine antigen copy number per tumor cell, cellular MFI was compared with a linear plot of bead MFI versus the number of PE molecules per bead. All staining was performed in 100 µl FACS buffer (PBS + 2% BSA). T cells were phenotyped with: anti-CD3 (BioLegend, clone HIT3a, PB), CD4 (BioLegend, clone SK3, FITC), CD8 (BD Biosciences, clone RPAT8, BUV395), biotinylated CD22 protein (Sino Biological, for CAR detection) and SA-PE (BioLegend). NK92, un-transduced PBMCs and CD22 CAR-transduced PBMCs were phenotyped with: anti-NKG2D (Biolegend, clone 1D11, APC), DNAM-1 (Biolegend, clone 11A8, APC), NKp30 (Biolegend, clone P30-15, PE), Nkp44 (BD Biosciences, clonep44.8.1, PE), NKp46 (Biolegend, clone 9E2, PE), TRAIL (Biolegend, clone RIK-2, PE), FasL (BD Biosciences, clone NOK-1, APC), KIR2DL1/DS1 (Beckman Coulter, catalog A09778, PE), KIR3DL1/DS1 (Beckman Coulter, catalog A60795, PE), NKG2A (Biolegend, clone S19004C, PE), ICAM1 (Biolegend, clone HA58, PE), ICAM2 (Biolegend, clone CBR-IC2/2, PE), LFA-1 (Biolegend, clone m24, APC), CD56 (BD Biosciences, clone R19-760, PE).

### CAR-T and Leukemia Cell Co-Culture and Separation

Anti-CD22 CART cells and leukemia targets (Raji, NALM6 and REH) were cultured at an effector to target ratio (E:T) of 4:1, 2:1, 1:1 or 0.5:1 for 24 hours with or without bryostatin, at which time CD19 and CD22 expression levels were quantified. To assess surviving leukemia target cells, co-cultures from the 1:1 ratio were harvested at 24 hours, cell populations separated by CD3-positive immunomagnetic bead selection (Stemcell Technologies), depletion verified by flow cytometry and CD3 negative cells (leukemia) cultured over time to assess antigen expression.

### Cut and Tag Analysis

Fresh cells (2x10^5^ to 5x10^5^ per treatment) were harvested and washed twice in 1.5 mL wash buffer (20mM HEPES pH 7.5; 150mM NaCl; 0.5mM Spermidine (Sigma S2501); 1× Protease inhibitor cocktail, Roche), and Cut&Tag libraries generated, following the protocol “Bench top CUT&Tag V.2” (22).

Cut and tag DNA libraries were sequenced on a HiSeq instrument (Novogene, Sacramento, CA), paired-end 150, with read depth of 17M per sample. The quality of sequencing data was checked by FastQC (23). FastQC: A Quality Control Tool for High Throughput Sequence Data). Sequencing adaptors identified and trimmed by TrimGalore [Trim Galore (RRID : SCR_011847)]. Sequencing reads were aligned to the UCSC Hg38 using the Bowtie2 package (24). Alignment results were normalized by the RPKM (Reads Per Kilobase of transcript, per Million mapped reads) method and methylation heatmaps around gene regions were plotted by DeepTools2 (25). Peak calling analysis was done by SEACR (26). Normalized bigwig results were visualized in UCSC genome browser. Differential peak analysis was done by DESeq2 (27) and peaks were annotated by GSCA (Ji Z and Ji H (2014), GSCA: Gene Set Context Analysis. R package version 1.4.0.).

### Cytolysis and Inhibition Assays

5 x 10^3^ target cells (Raji-Luc, NALM6-Luc, REH-Luc or K562-Luc) were co-cultured with UTD control or anti-CD22 CAR-T cells at various effector to target ratios (16:1, 8:1, 4:1, and 2:1) in 96-well plates and incubated overnight at 37°C, 5% CO_2_ in 100 μL of complete RPMI medium without cytokines. Twenty-four hours later, 100 μL of SteadyGlo reagent (Promega) was added to each well and incubated for 10 minutes at room temperature followed by quantification of luminescence using an Enspire plate reader (Perkin Elmer). The luminescence was captured as counts per second (CPS) for each experimental well containing the indicated E:T ratio (sample CPS), target cells alone (target CPS) and tween-20 treated target cells (negative CPS). Percent specific lysis presenting luciferase reduction was calculated as: (1- (sample CPS-negative CPS)/(target CPS-negative CPS)) x 100%.

For ligand-based cytolysis blocking assays, 5 x 10^3^ target cells (NALM6-Luc or REH-Luc) were plated in a 96-well plates in 50uL complete RPMI medium. Recombinant protein (DNAM-1-his, Acro Biosystems, DN1H52H6; NKG2D-his, Acro Biosystems, NKDH5245; NKp30, Acro Biosystems, NC3H5228) or anti-ICAM1 antibody (Biolegend, 322721) was added to target cells at 10ug/mL and incubated at 37°C for 30min. 5 x 10^4^ effector cells (NK92, UTD or CD22 CART) were added to target cells and treated with 1nM Bryostatin at 37°C overnight.

### Reverse Transcription Droplet Digital PCR (RT-ddPCR)

Cells from each condition were collected and RNA was isolated by RNeasy mini kit (Qiagen, Catalog#74104). RNA quality was checked by high sensitivity RNA ScreenTape assay (Agilent, 4200). RNA quantity was determined by Qubit RNA RS kit (Thermo Fisher). RNA samples were mixed with one step RT-ddPCR advanced kit for probes (Bio-Rad), together with ddPCR GEX primer/probe for CD19 or CD22 (Bio-Rad) in a 96 well plate to generate RT-PCR reaction mix. Reaction droplets were generated by QX200 AutoDG droplet generator, PCR reaction was performed by C1000 Touch Cycler (Bio-Rad). RT-PCR droplets were read by QX200 droplet digital PCR system and data was analyzed using Quantasoft (Bio-Rad).

### Western Blot

One and a half million cells from each treatment were washed twice in cold PBS, lysed in 100 ul cold RIPA buffer (Bio-Rad) containing protease inhibitor cocktail (Roche). The lysate was incubated for 1 hour on ice, pelleted at 15000 RPM at 4°C for 20 min, and supernatants collected and mixed with 200 µl Laemmli Sample Buffer (Bio-Rad), then boiled for 3 min at 100°C. Protein concentrations were determined by Nanodrop and 20µg of each sample resolved by PAGE and proteins transferred to 0.45 μm nitrocellulose transfer membrane (Bio-Rad) and probed with primary antibodies against CD19 or CD22 with β-actin (Odyssey Li-Cor, Lincoln NE) overnight at 4°C, and secondary IRDye 800CW antibody at room temperature for 1 hour. Bands were visualized and quantified on an Odyssey imaging system with Image Studio lite software (LI-COR). Relative band intensity of CD19 and CD22 was calculated and normalized to β actin.

### Statistical Analyses

Plots show average of three replicate wells, standard deviation, and p-value as calculated by nonparametric t test, unless otherwise noted. All plots and analyses were analyzed using Prism software (v. 9.2.0, GraphPad Software, LLC) and are representative of three experiments, unless otherwise noted.

## Results

### Impact of Differentiation Agents on CD22 and CD19 Surface Expression

**Figure 1:**
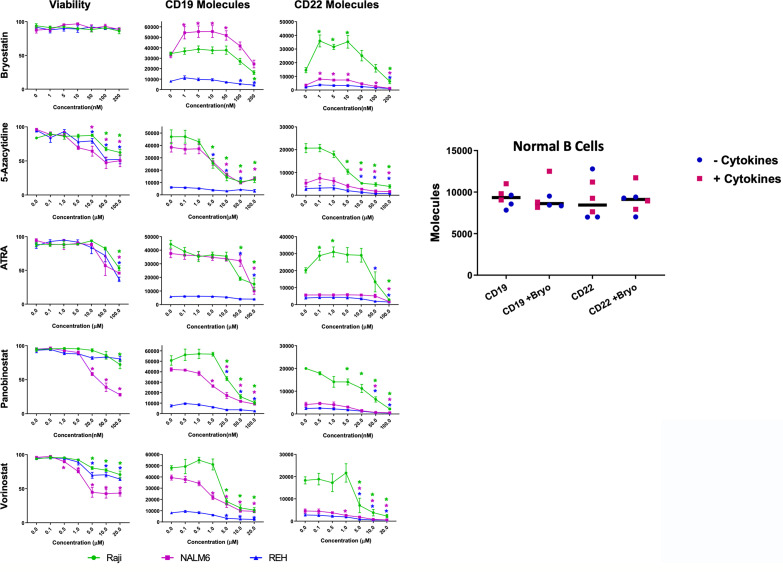
Viability and Surface Expression of CD19 and CD22 in treated B-cell leukemia cell lines and normal B cells. Left panel: Epigenetic modifiers/differentiation agents (Bryostatin, 5-Azacytidine, ATRA, Panobinostat or Vorinostat) were added at increasing concentrations (x-axis, as indicated) to the culture media of B cell lines (Raji-green circle, NALM6-magenta square, REH-blue triangle) for 48 hours (or 24 h for bryostatin). Following drug exposure, cell viability was calculated and plotted (column 1). Each agent adversely affected viability as concentration increased, except for bryostatin. Surface expression of CD19 (column 2) and CD22 (column 3) in leukemia cell lines was qualified by flow cytometry using Quanti-Brite PE beads. Average of triplicate wells is shown, values differing from untreated controls are indicated, * indicates p<0.05. Right panel: Expanded peripheral blood B cells from three donors, cultured on CD40L expressing feeder cells in media supplemented with (squares) or without (circles) B cell growth factors (IL-2, IL-4, IL-21, BAFF, see *Materials and Methods*), were tested for changes in cell surface expression induced by bryostatin. The number of CD19 and CD22 molecules differed between donors to a degree, but was not significantly impacted by bryostatin, paired t-test p>0.05, grand median, solid bar, shown for reference.

The modulation of CD22 expression levels on the surface of leukemia cells is of great interest to the immunotherapy field. To explore mechanisms to increase CD22 expression we tested whether differentiation agents or epigenetic modifiers that are well-studied in human clinical trials are able to impact the expression of CD22 on the surface of model cell lines as well as normal B cells. The B cell leukemia lines tested were the Burkitt lymphoma cell line Raji, and the B cell acute lymphocytic leukemia (ALL) cell lines NALM6 and REH. Panobonistat was tested for impact on cell viability and target antigen expression from 0.5 to 100 nM, at 48 hours. No impact on viability was seen up to 5 nM (**Figure 1**). No increase in CD22 expression was seen in this concentration range (Summarized in **Supplementary Table 1**). Vorinistat (SAHA) was also tested at 48 hours, at concentrations ranging from 0.1 to 20 uM. Impact on viability was seen at 1 or 5 uM, and at or below these ranges no increase in CD22 expression was seen. ATRA was tested between 0.1 and 100 uM at 48 hours, and no impact on cell viability was seen at 10 uM or below. Notably, ATRA increased CD22 expression on Raji cells, while NALM6 and REH levels remained constant. 5-Azacytidine was also tested at 48 hours at concentrations between 0.1 and 100 uM. No impact on viability was seen at 5 uM or below. While a slight rise in CD22 expression was seen at 0.1 mM 5-Azacytidine, this difference did not reach statistical significance. Because of the rapidity of effects seen with bryostatin, experiments were carried out for 24 hours. Bryostatin has no impact on cell viability from 1 nM up to 200 nM, and increased CD22 expression in each cell lines tested, although the change in REH was not statistically significant. Assays were also carried out in a similar manner to assess the impact of each agent on the number of surface CD19 molecules expressed per cell. CD19 was far less amenable to modulation by epigenetic or differentiation agents. Only with bryostatin, and only in the NALM6 cell line, were statistically significant increases noted.

To explore the impact of bryostatin on CD22 and CD19 expression on normal B cells, B lymphocytes were purified by negative selection (untouched) and cultured on a CD40L-expressing feeder cell line, with or without the supporting cytokines, IL-2, IL-4, IL-21 and BAFF, as reported by Su et al., for seven days (18). Expanded B cells were cultured in the presence of 1 nM bryostatin for 24 hours and the number of CD19 and CD22 molecules per cell analyzed. Expression of CD22 and CD19 on the expanded normal B cell population was not affected by bryostatin, **Figure 1**. This implies that the response of Raji more closely resembles normal B cells, in keeping with the more developmentally mature status of Burkitt lymphoma in comparison to pre-B-ALL. We further explored the mechanism by which bryostatin impacted the level of CD22 antigen expression by quantifying the RNA and protein.

### Up-Regulation of CD22 by Bryostatin Includes Minor Increases in Transcriptional Activation

**Figure 2:**
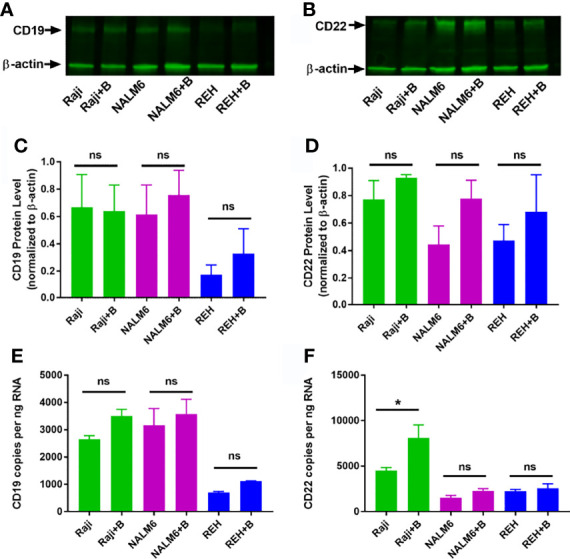
Total RNA and protein levels of CD19 and CD22 in bryostatin treated leukemia cell lines. Western blot analysis of **(A)** CD19 and **(B)** CD22 protein expression in Raji, NALM6 and REH cell lines with (+B) or without bryostatin treatment. **(C)** CD19 and **(D)** CD22 band intensity from three independent experiments was quantified and normalized to β-actin. For each line, treated and non-treated groups were compared. There was no significant difference (ns) between groups of at the protein level. RNA levels for **(E)** CD19 and **(F)** CD22 were quantified by ddRT-PCR. CD19 and CD22 copies per ng RNA were calculated and analyzed. Significant differences between treated and untreated groups were seen for CD22 in Raji cells (*p < 0.05*). *p < 0.05. ns, not significant.

Bryostatin had the broadest effect (with respect to degree of increase in CD22 and consistency across cell lines) on CD22 surface expression. We therefore sought to establish if this effect was due to a concomitant increase in total CD22 protein, as well as measuring the amount of CD22 mRNA. Although bryostatin appeared to increase the amount of total protein for both CD22 and CD19, these differences were not statistically significant when assessed by Western blot, **Figure 2**. Likewise, when the amount of mRNA encoding these surface markers was quantified, no significant differences were seen, except for CD22 expression in Raji.

In addition to the known modulation of protein kinases (PKC delta and epsilon) and c-Jun, we sought to determine if bryostatin induces changes in the epigenome of treated cells (28). This would extend the known effects of this agent to include modulation of global gene expression programs, and perhaps identify specific alterations. Cut&Tag analysis (Cleavage Under Targets and Tagmentation), developed by the Henikoff lab at the Fred Hutch, goes beyond ATACseq, in that specific epigenetic modifications of histones, as determined by specific antibody cleavage sites, are measured and characterized (22, 29). Increased H3K4me3 and H3K4me2 signal (trimethylation or demethylation of lysine 4 on the histone H3, associated with activation of transcription from nearby promoters) or the opposing H3K27me2 (dimethyl state of lysine 27 of histone H3, associated with inactivation of transcription) marks can be readily visualized by mapping resultant amplified segments. Global alignment of transcriptional start sites identified by Cut and Tag demonstrates that our analysis compares numerous bryostatin-induced changes in gene expression, and that bryostatin treatment in and of itself did not profoundly change the net transcriptional activity of the treated leukemia cell lines (**Supplementary Figure 1**). For CD22, small, but statistically significant, increases in reads for H3Kme2 in Raji cells, and for H3Kme3 for all three lines (Raji, NALM6, REH) were seen with bryostatin treatment (**Supplementary Figure 2**). The only significant change for CD19 was seen in Raji cells, and only for H3Kme3 (**Supplementary Figure 3**). Although there are slight increases in mRNA and total protein expression, and bryostatin does have measurable epigenetic effects, these are unlikely to account for the rapid increase in target antigen expression induced by bryostatin over 24 hours.

### Coculture of Leukemia Cells With CD22 CAR-T Decreases On-Target and Off-Target Antigen Expression

**Figure 3:**
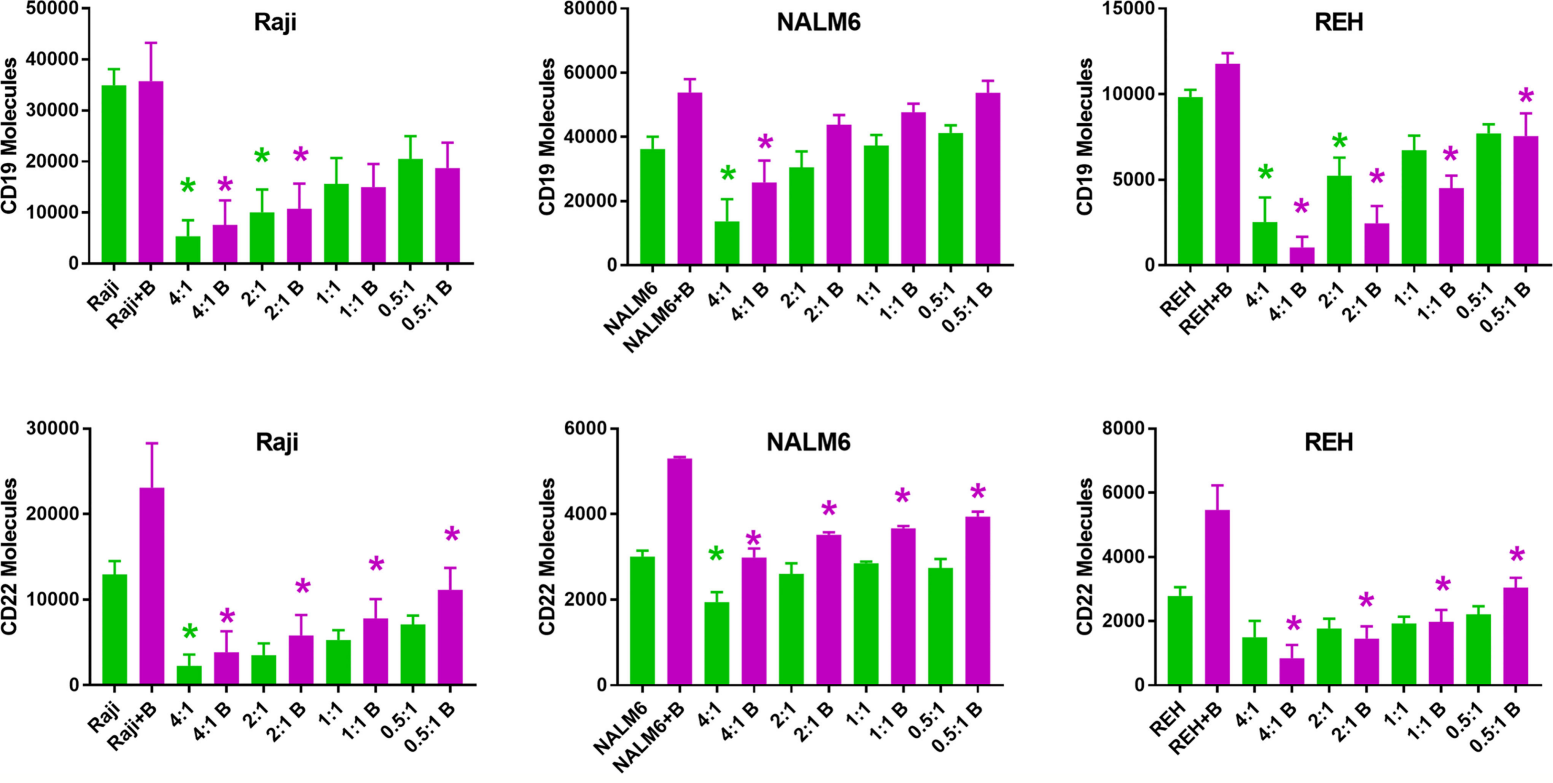
Surface expression of CD19 and CD22 upon co-culture with anti-CD22 CAR-T. Using Quanti-Brite analysis, the number of CD19 and CD22 molecules (y-axis) on the surface of Raji, NALM6, and REH cell lines was quantified, following co-culture with CD22 CAR-T, at the indicated effector to target ratios, x-axis. The leftmost pair of columns quantifies surface expression on untreated cell lines. Significant differences from control are shown *p < 0.05. The x-axis lists the cell line tested, exposure to bryostatin (B, magenta bars) or CD22 CAR-T alone (green).

The observation that relapsed disease is associated with a lower expression of CD22 antigen on the leukemia cell surface led us to explore the temporal interactions between CAR-T cells and leukemia cell line targets in the presence of bryostatin. Using a range of effector (CD22 CAR-T) to target (leukemia line) ratios (E:T) we found that the co-incubation of CAR-T with leukemia cell lines induces a profound decrease in the number of cell surface antigens expressed on the cell surface (**Figure 3**). The assay was carried out by culturing leukemia cells for 24 hours in the presence of 1 nM bryostatin for 24 hours, followed by the overnight addition of CD22-specific CAR-T for another 24 hour period, again in the presence of bryostatin. At the concentration used, bryostatin does not impact CAR-T activity (not shown). As expected, CD22 CAR-T induced profound and rapid down-regulation of CD22 antigen expression on the leukemia cell surface. Surprisingly, this effect was also seen when the levels of CD19 were analyzed on the leukemia cell surface, **Figure 3**. Thus, CD22 CAR-T cells rapidly down-modulate not only CD22 but also CD19. The effects were seen with or without bryostatin addition. However, including bryostatin did have an effect on the net amount of antigen down-modulation, in that moderately higher levels of antigen expression were noted for both targets during CAR-T co-culture. Thus, in a short-term assay, bryostatin impacts target antigen expression. Overall, co-incubation with CAR-T decreases CD22 and CD19 surface antigen expression on leukemia cells surviving CD22 CAR-T co-culture. Antigen expression was somewhat higher in Raji and NALM 6, and somewhat lower in REH treated with bryostatin. This informs us that inclusion of brysotatin, most clearly for NALM6, keeps target antigen expression at a higher level even while undergoing CAR-induced antigen down-modulation, and thus may aid in immune elimination.

### Down-Regulation Occurs Rapidly, and Reverses Rapidly

**Figure 4:**
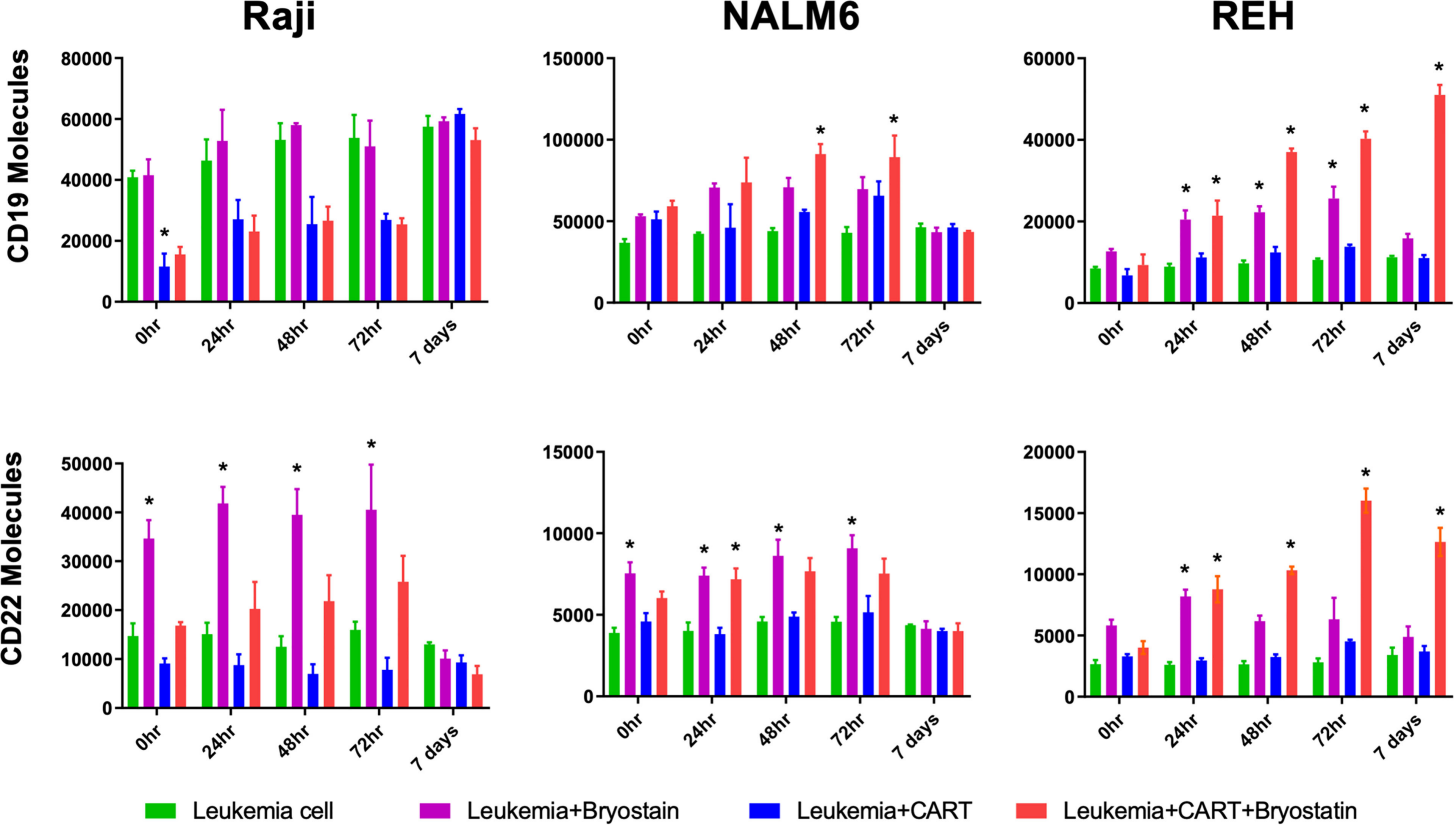
Surface expression of CD19 and CD22 following bryostatin wash-out and CAR-T removal. After overnight culture with anti-CD22 CAR-T, the number of cell surface proteins was quantified using Quanti-Brite analysis, average of triplicate wells and standard deviations are shown. 0 hr, x-axis, is after the overnight culture, and each time point represents cell surface proteins on the surface of untreated Raji, NALM6, or REH (Leukemia cell, green bars), treated with bryostatin alone (Leukemia + Bryostatin, magenta bars), treated with CAR-T alone (Leukemia+CART, blue bars), or treated with both CAR-T and bryostatin (Leukemia+CART+Bryostatin, red bars), at the time points listed, x-axis. Significant differences from leukemia alone are shown *p < 0.05.

**Figure 5:**
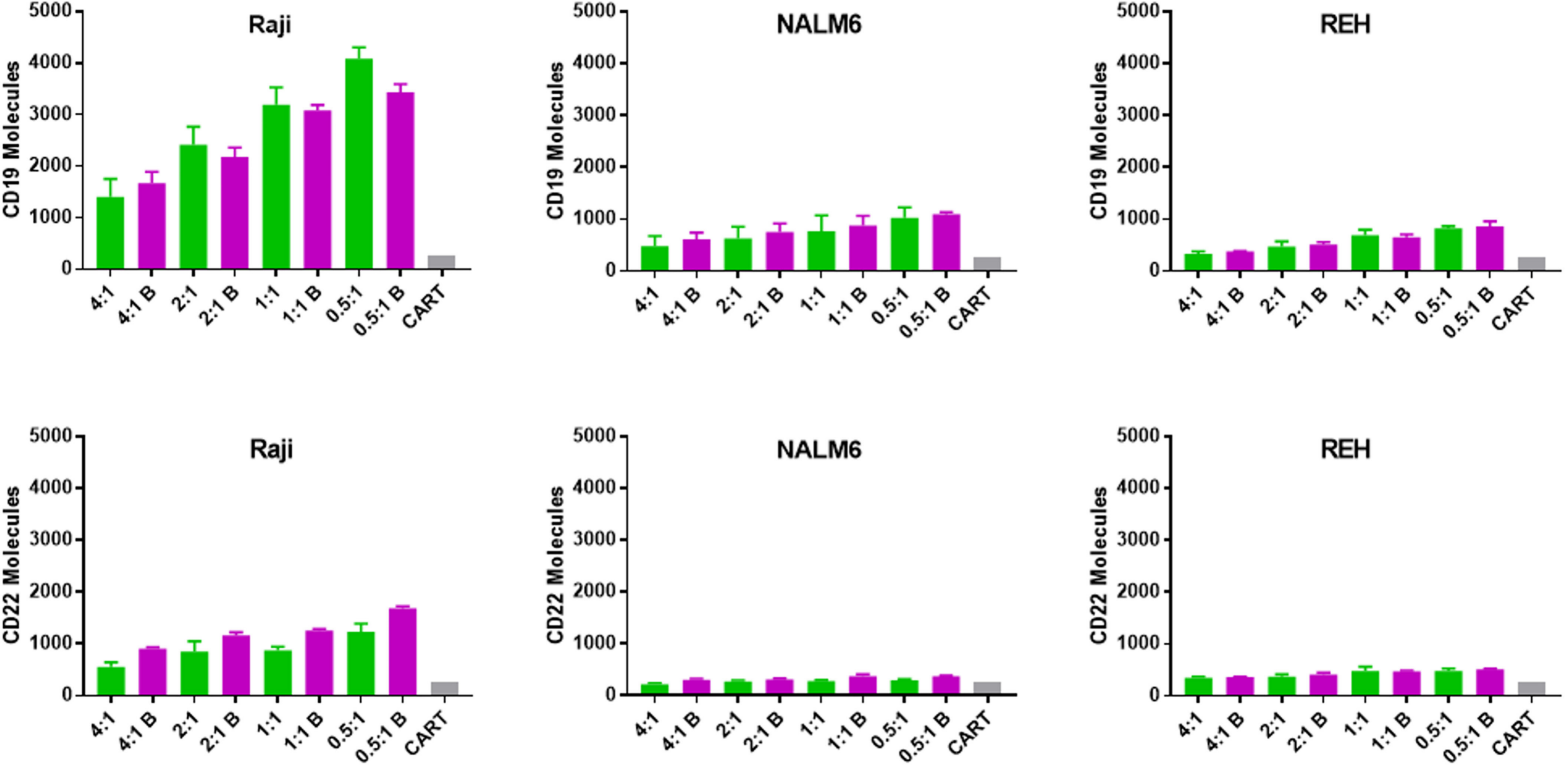
Transfer of CD19 and CD22 to CD22 CAR-T following overnight culture with leukemia cell lines. The number of CD19 and CD22 molecules acquired by anti-CD22 CAR-T (trogocytosis) was quantified using Quanti-Brite analysis, as per **Figure 3**, however in this case the T cells were analyzed. Average antigen expression and standard deviation are shown. Background signal is shown as a gray bar for each subgroup (CART). The x-axis lists the anti-CD22 CAR-T to leukemia cell ratio (E:T) used for each condition and indicates if the leukemia line had been treated with bryostatin (B, magenta bars).

To determine if CAR-T-mediated CD22 on-target and off-target antigen modulation was a lasting effect, CAR-T and leukemia cells were separated following overnight co-culture using anti-CD3 immunomagnetic beads, and leukemia cells cultured alone in fresh media. Following removal of CD22-specific CAR-T cells, cultured leukemia cell lines demonstrated differential re-expression of CAR target antigens, **Figure 4**. Raji cells co-cultured with CD22-CAR-T took more than 3 days to fully recover CD19 expression from CD22 CAR-T exposure, yet this recovery was complete. As expected bryostatin markedly upregulated CD22 on Raji cells, this increased level persisted to day 3, and returned to original levels by day 7. While CD22 CAR-T reduced CD22 levels for at least 3 days, this effect was markedly reversed by bryostatin. Thus, with Raji targets, bryostatin has a decidedly beneficial impact on the upregulation of CD22. NALM6 showed a similar preservation of both CD19 and CD22 upregulation following CD22 CAR-T co-culture in the presence of bryostatin. At the E:T ratio evaluated, no large down-regulation of CD22 expression was seen due to CD22 CAR pressure. This requires the higher E:T presented in **Figure 5**. The REH cell line displayed an unexpected result. Immediately following separation from CAR-T and at 24 hours, bryostatin alone and bryostatin and CD22 CAR-T had increased CD19 and CD22 expression. Either bryostatin treatment alone or CD22 CAR-T treatment had no long-term effect, as by day 7 expression levels returned to those of untreated REH. However, treatment or REH with bryostatin and CD22 CAR-T cells resulted in prolonged upregulation of both CD19 and CD22 expression. This result will be explored in future studies, and implies an interesting additive effect.

### Trogocytosis Is Unlikely to Play a Major Role in Antigen Down-Regulation

One well-described mechanism for altering or sharing cell surface antigen expression is trogocytosis, defined as transposition of cell membrane or cell membrane proteins between cells during cell-cell interactions (30). We tested if CAR-T cells were able to acquire either on-target or off-target cell surface antigen upon co-culture with leukemia cells, **Figure 5**. When CAR-T cells specific for CD22 were analyzed for either CD22 or CD19 acquisition following co-culture with leukemia target cells, CD19 appeared to transfer more readily to the CAR-T cell surface than CD22. This likely reflects the relative increased abundance of CD19 on the membrane of the leukemia cell. Clearly, trogocytosis is not limited to the CAR target antigen, as both CD19 and CD22 were transferred to the T cell surface. Moreover, this supports the original definition of trogocytosis, the transposition of a membrane patch, as opposed to single protein transfer. Importantly, this effect was not uniform across the leukemia cell targets. While Raji cells appeared to readily transfer membrane (and thereby CD19 and CD22 expression on CAR-T), this effect was quite limited in NALM6 and REH cells and unlikely to drive the loss of target antigen expression at the cell surface we measured.

### CD22 CAR-T and NK-92 Activity Against Leukemia Depends on the Leukemia Cell Type and the Effects of Bryostatin Treatment

**Figure 6:**
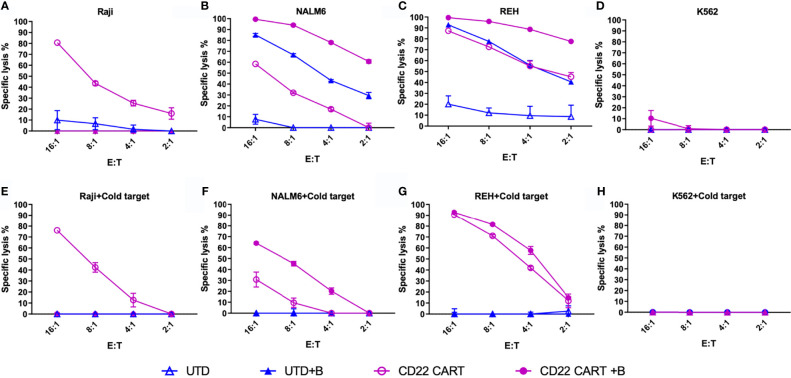
Anti-CD22 CAR-T mediated cellular cytotoxicity (CTL) of bryostatin-treated leukemia. **(A–D)** Average lysis from triplicate wells for four cell lines (Raji, NALM6, REH, and K562) by anti-CD22 CART (CD22 CART, open circle) or un-transduced T cells from the same donor (UTD, open triangle), treated with bryostatin (+B, closed shape) or untreated (open shape), a the E:T ratios listed on the x-axis. **(E–H)** Assay tested in parallel including cold-target inhibition (addition of K562 at a 30:1 E:T ratio). Representative results for T cells from 3 donors are shown, each data point showing the average and standard deviation from three replicate wells.

Our motivation for studying the down-modulation of target antigens was to explore the effect of epigenetic modifiers on these changes, and to determine their overall effect on leukemia cell cytolysis. When cytolytic assays were caried out following pre-treatment of leukemia target cells with bryostatin, we found differential effects according to the cell line analyzed, **Figures 6A–D**. Without bryostatin, we found that increasing E:T ratios resulted in increased cytolysis for all cell lines, with the exception of K562, an antigen negative leukemia included as a control for NK cell-like activity. For the ALL lines NALM6 and REH, the killing of leukemia targets mediated by CD22 CAR-T was greatly enhanced by bryostatin. However, we also saw an increase in the killing of these ALL lines mediated by untransduced/activated (UTD) T cells induced by bryostatin. Thus, treatment of leukemia targets (as indicated by +B in **Figure 6**) with bryostatin had a profound effect on cell-cell killing mediated by activated T cells in general, implying that non-CAR-T specific killing mechanisms were invoked. To the contrary, Raji cells showed the opposite effect. Although bryostatin does indeed increase the target antigen number on the cell surface (**Figure 1**), bryostatin treatment results in a marked inhibition of cellular cytotoxicity. A classic cellular immunology technique to block non-antigen dependent (usually NK-associated) killing is called “cold-target inhibition” (31–33). In this technique, used to differentiate between receptor-mediated ADCC, NK cell activity, and “natural” cytolysis by other immune cell subtypes, a 30:1 excess of unlabeled (in this case luciferase non-expressing) K562 cells are added into the cellular cytolysis assay, **Figures 6E–H**. Cold target inhibition had no effect in the Raji cytolysis assay. This indicates that CD22 CAR-T activity against Raji is strictly driven by the CAR, and not other target antigens initiating susceptibility to UTD-mediated killing. For the REH cell line, bryostatin treated cells upregulated ligands that were recognized by activated T cells, i.e. strong UTD-mediated killing was induced. When unlabeled K562 were added to the killing assay for cold-target inhibition, non-specific killing by non-CAR expressing activated T cells (UTD) was blocked, **Figure 6G**. This indicates that induction of a set of classical NK ligands on REH was responsible for the UTD-mediated killing. The same effect was seen when CD22 CAR-T and NALM6 were co-incubated, **Figure 6F**, although bryostatin appeared to have a more pronounced effect. Thus, bryostatin treatment induces B-ALL sensitivity to both CAR-T specific and non-specific killing mechanisms. The ability to block these effects with an excess of unlabeled K562 cells demonstrates that activation of T cells to produce CAR-T induces an NK-like activity. However, the ligands to detect this activity requires the ALL to first be activated by bryostatin. This could thus be classified as a bryostatin-induced off-target/on-tumor activity. We have termed this “CAR T-cell antigen-non-specific killing” or CTAK, to differentiate it from NK- or LAK-mediated killing. It requires both the induction of new targets on the leukemia and the ligands expressed on highly activated T cell populations, such as those induced by CAR-T production.

**Figure 7:**
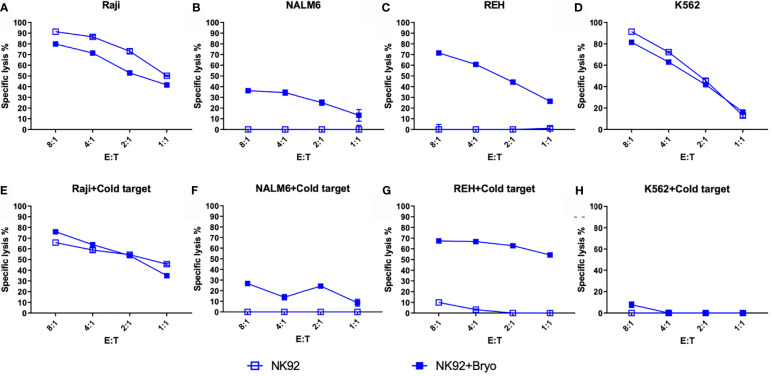
NK92-mediated cellular cytotoxicity of bryostatin-treated leukemia. **(A–D)** Average lysis from triplicate wells for four cell lines (Raji, NALM6, REH, K562) mediated by NK92 cells using untreated (open square) or bryostatin-treated (closed square) targets at the E:T ratios listed on the x-axis. **(E–H)** Assay tested in parallel including cold-target inhibition. Representative results, average of triplicate wells and standard deviation, from 3 independent experiments are shown.

**Figure 8:**
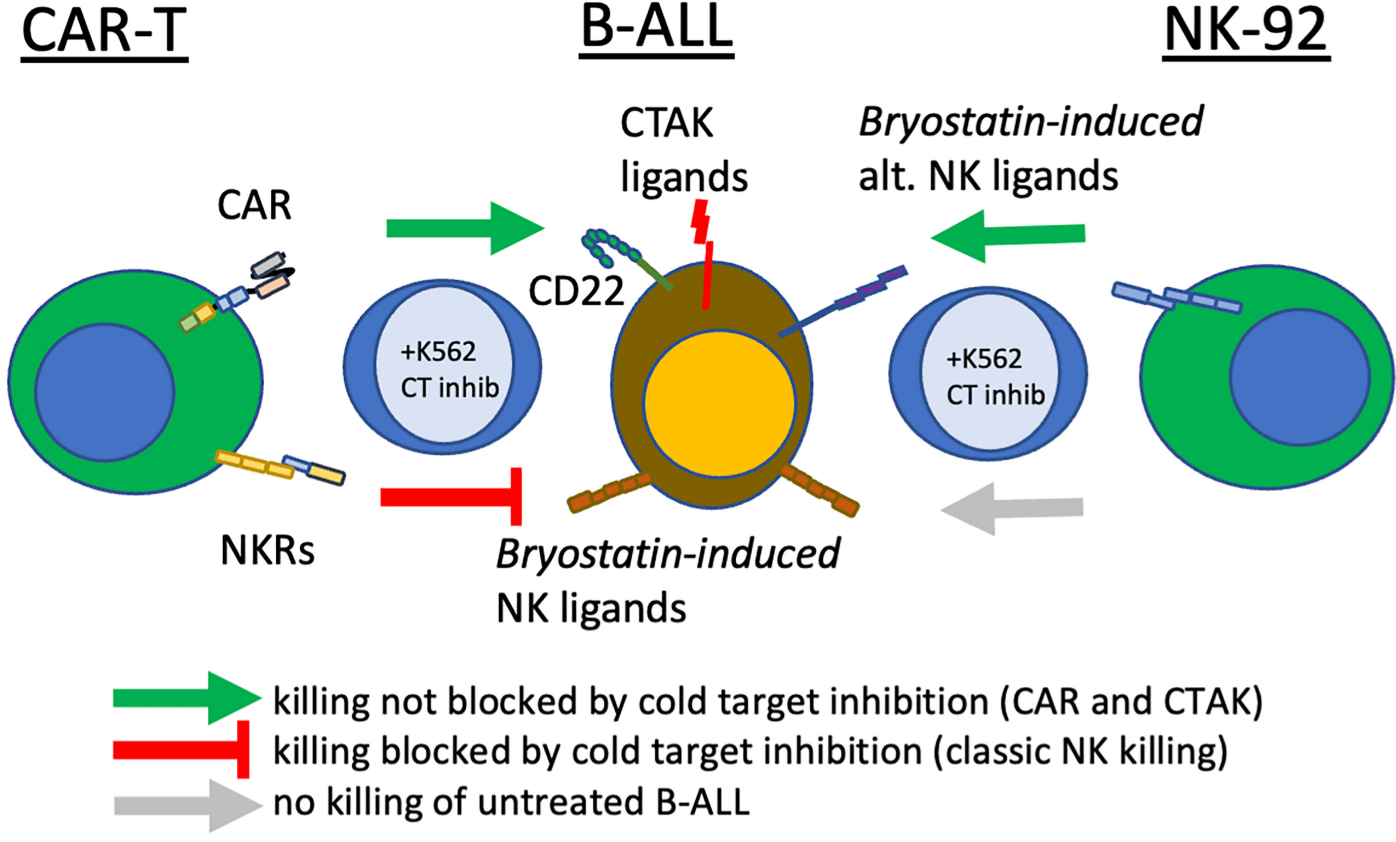
Bryostatin treatment reveals multiple pathways that CAR-T cells use to eliminate leukemia. In the center of the diagram, pre-B ALL cells are illustrated, displaying the CAR-T target antigen, CD22, innate immune receptor ligands induced by bryostatin that are recognized by activated T cells (Bryostatin-induced NK ligands) and ligands recognized by T cells that have been: a) sensitized by CAR-T production, b) bryostatin-induced, and c) not blocked by cold-target inhibition (CTAK, CAR-T cell non-antigen-specific killing). Also shown are a non-overlapping set of alternative innate immune receptor ligands that are recognized by NK92 upon bryostatin-treatment (right-most effector cell). Cold-target inhibition does not affect NK92 or CD22-specific CAR-T killing. Cold-target does decrease killing evidenced by activated T cells (UTD), but incompletely for CTAK-mediated killing. Green arrows indicate successful cytolysis and blunt red arrow indicates killing impacted by K562-mediated cold target inhibition (classic NK killing).

To further explore the activity of NK cells against bryostatin-treated B cell leukemia cell lines, we tested the NK92 cell line in direct cytolysis assays, **Figure 7**. Use of NK-92 cells avoids donor-to-donor variability and NK culture condition concerns. NK92 are currently being tested natively or modified with CARs in clinical trials, and may represent a complimentary treatment option to CAR-T (34, 35). Our data demonstrate that the Raji cell line is effectively lysed by NK92, and that this lysis is not impacted by the presence of K562 cold-target inhibition, **Figure 7E**. As the control experiment with K562 demonstrates (**Figures 7D, H**), cold-target inhibition completely abrogates cytolysis of the self-same target. The results with the pre-B ALL cell lines were unexpected, in that there was no lysis of ALL by NK-92 without bryostatin treatment. Moreover, once NK-92 ligands were induced, these target antigens were not blocked by K562-based cold target inhibition. These results indicate that bryostatin induces two classes of targets for the innate immune system. Some are analogous to classic NK-targets (K562-like). Other leukemia expressed targets -while being recognized by NK-92- are not blocked by K562 cold-target inhibition, as illustrated in **Figure 8**. Our flow cytometric analysis of NK-92 is in agreement with previous studies, demonstrating strong CD56, as well as NKG2D, KIR2DL3, NKp30, NKp44, NKp46, and Fas staining; and low staining for NKG2C, KIR2DL1, FasL, DNAM, and KIR3DL1 (**Supplementary Table 2** and **Supplementary Figure S7**). Published analysis by others of potential NK targets expressed on K562 demonstrated very high expression for ICAM1, ICAM2, NKp30, HLA-F, MIC-A, ULBP2, ULBP3, CD48, CD80, CD112 (PVRL2/NECTIN2), CD155 (PVR) (36), thus providing multiple candidates whose expression, either singly or in combination, may be responsible for cold-target inhibition.

### EBV Latency Reactivation in Not the Major Driver of CD22 CAR-T Resistance to Cytolysis in Bryostatin-Treated Raji Cells

**Figure 9:**
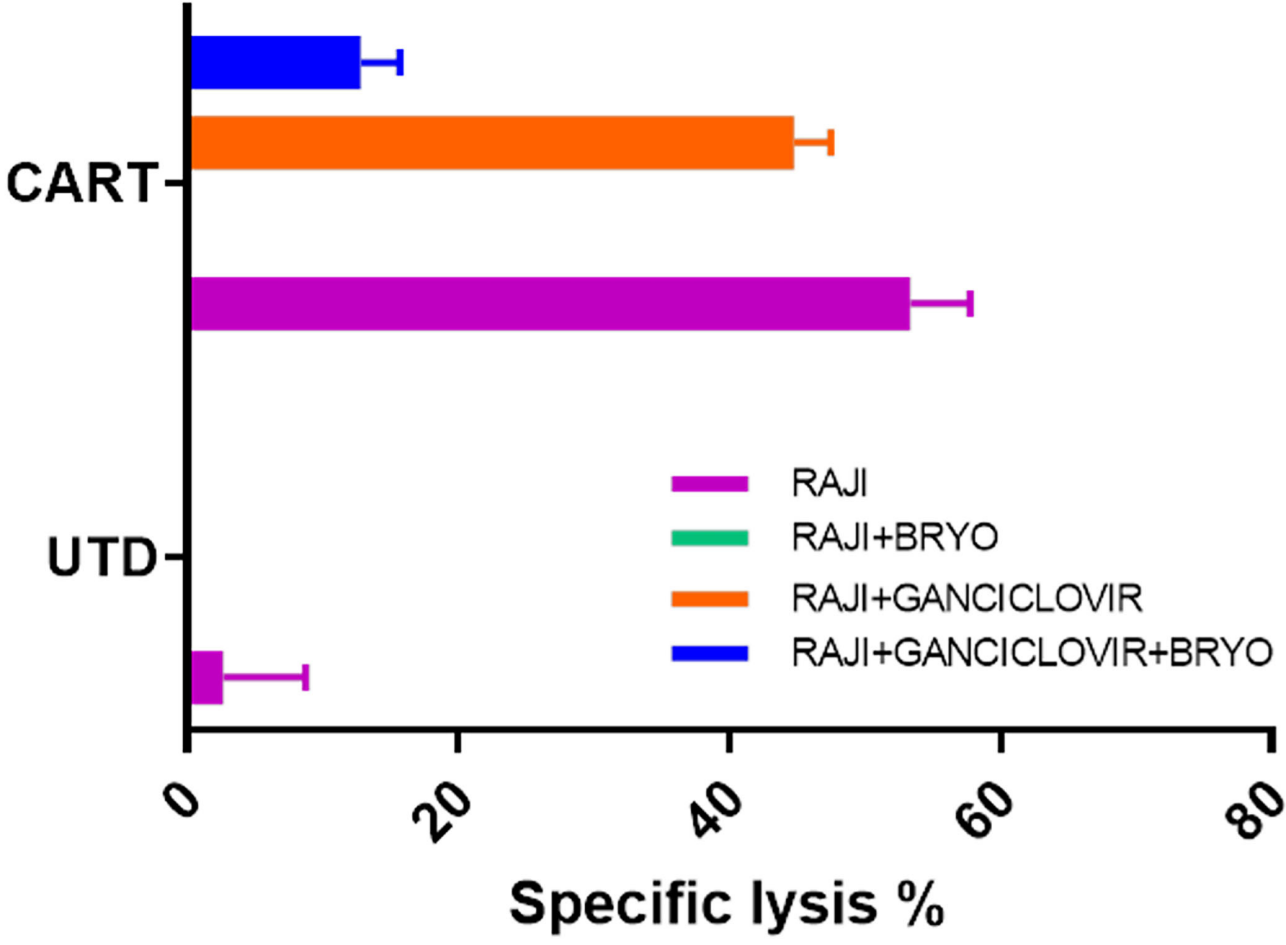
Impact of EBV lytic cycle inhibition on CD22 CAR-T mediated killing of bryostatin-treated Raji leukemia cells. Raji cells were cultured for 2 weeks in the presence or absence of 15 uM ganciclovir (+Ganciclovir in legend), and for the final day of culture bryostatin was added where indicated (+Bryo in legend). Treated cells were then used as targets in CTL assays using anti-CD22 CAR-T (CART) or untransduced T cells (UTD) as effector cells. Average cytolysis of 3 replicate wells is plotted for each condition. Results are representative of three independent experiments.

**Figure 10:**
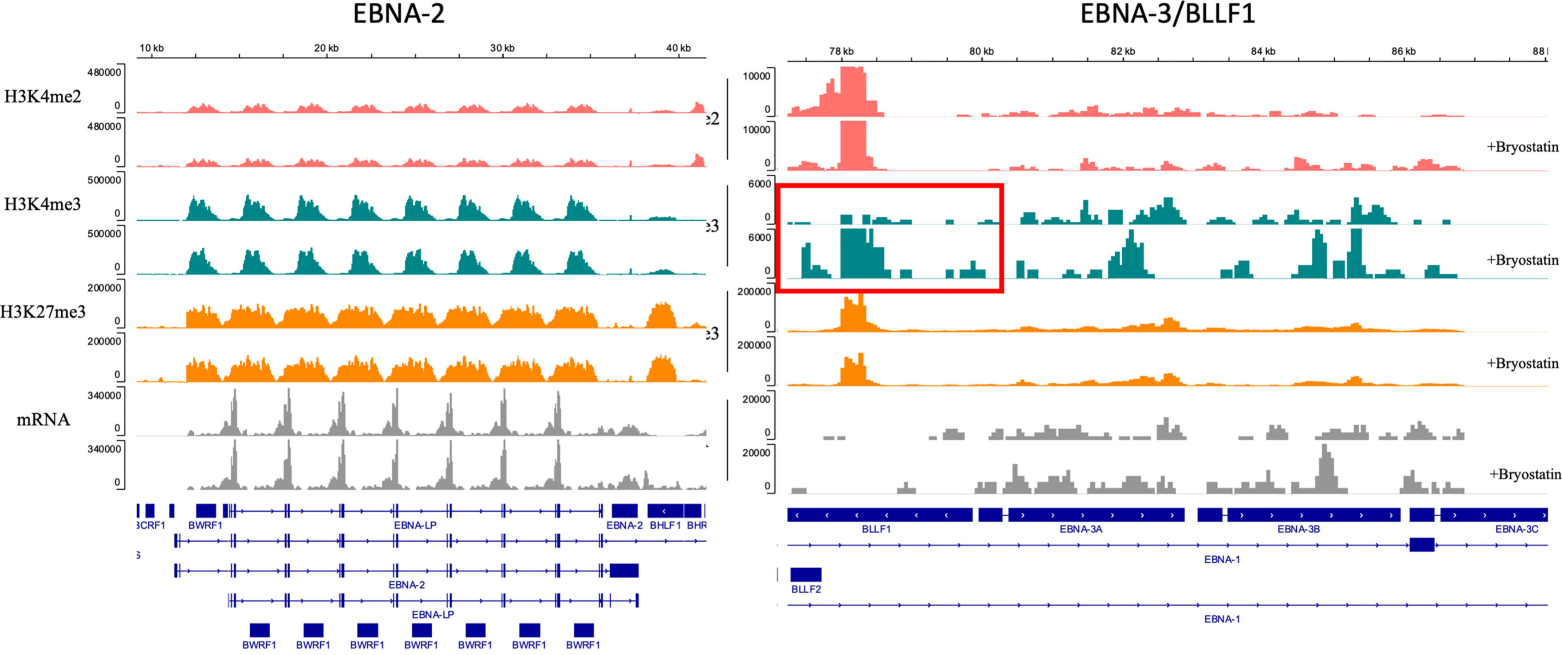
Epigenetic modulation of the EBV genome in Raji leukemia cells mediated by bryostatin. Sequenced reads for transcriptional activators of EBV latency, left panel) EBNA-2 and EBNA-LP, and right panel) EBNA-3A,-3B,-3C and BLLF1; were analyzed by Cut&Tag analysis and mapped on the EBV genome for the presence of epigenetic modification of H3K4me2, H3K4me2, and H3K27me3 (y-axis). No reads were detected for the IgG control. Reads are presented as parallel samples for bryostatin-treated (+bryostatin) or untreated Raji. Shown below the immunoprecipitated Cut&Tag reads are total mRNA reads (in gray) displayed over the relevant portion of the EBV viral genome, shown at the bottom portion of the plot. Changes demonstrated for H4K4me3 reads for BLLF1 are indicated by the red square.

Raji is an EBV-positive Burkitt lymphoma cell line. Principal Component Analysis (PCA) of bulk RNAseq data demonstrated that Raji clusters closer to normal B cells in comparison to either REH or NALM6, reflecting its well-established more differentiated B cell status as a Burkitt lymphoma (not shown). We tested the impact of inhibiting EBV replication or activation by culturing Raji cells in ganciclovir for two weeks. Previous work demonstrated the requirement for this extended time of treatment to insure complete viral quiescence for B-LCLs (37). CD22 CAR-T lysed Raji cells efficiently while control UTD did not, **Figure 9**. Furthermore, treatment with bryostatin renders Raji cells resistant to CD22 CAR-T-mediated cytolysis. The addition of ganciclovir reversed bryostatin-mediated resistance to a small degree, and some restoration of killing by CD22 CAR-T was demonstrated. Thus, bryostatin-mediated modulation of latent EBV gene expression may in some part explain the induced resistance to CAR-T mediated killing. We examined epigenetic alterations in EBV latency-associated genes to see if these were altered by the addition of bryostatin. No changes in histone methylation were seen for the Epstein-Barr virus associated latency antigens EBNA1, EBNA2/EBNA-LP, LMP1 or LMP2, although a slight decrease in mRNA expression was noted for the latency membrane proteins (**Supplementary Figures 4**, **5**). Changes in canonical markers of EBV reactivation, Zta and Rta, or for LF1,2 or 3 were not seen (**Supplementary Figure 6**). Interestingly, when we examined the regulation of EBNA3 promoter regions by methylation we did not find any changes for EBNA 3A, 3B, or 3C, but did increased marks for H3K4me3 (indicating increased transcriptional activity) for BLLF1, **Figure 10**. Unlike the EBNA proteins which serves as transcriptional regulators, BLLF1 encodes the major viral surface glycoprotein gp350. The gp350 receptor is CR2/CD21. CD21 is expressed on both T and NK cells, and interacts in concert with other receptors to mediate either cellular activation or viral infection (38, 39). Thus, in searching for a potential explanation as to why bryostatin induces Raji resistance to CD22 CAR-T, we found small changes in latent EBV viral genome regulation, and a minor but detectable reversal of the bryostatin effect by ganciclovir. Taken together this indicates that while the latent EBV genome in Raji does play a role in immuno-evasion, and bryostatin partially reverses this effect, the majority of bryostatin-mediated immune-evasion is attributable to factors inherent in the Burkitt lymphoma genome itself.

### Increased Expression of Both Adhesion Molecules and NK Ligands Contributes to CAR T-Cell Antigen-Non-Specific Killing (CTAK), and CAR-T NK-Like Killing

**Figure 11:**
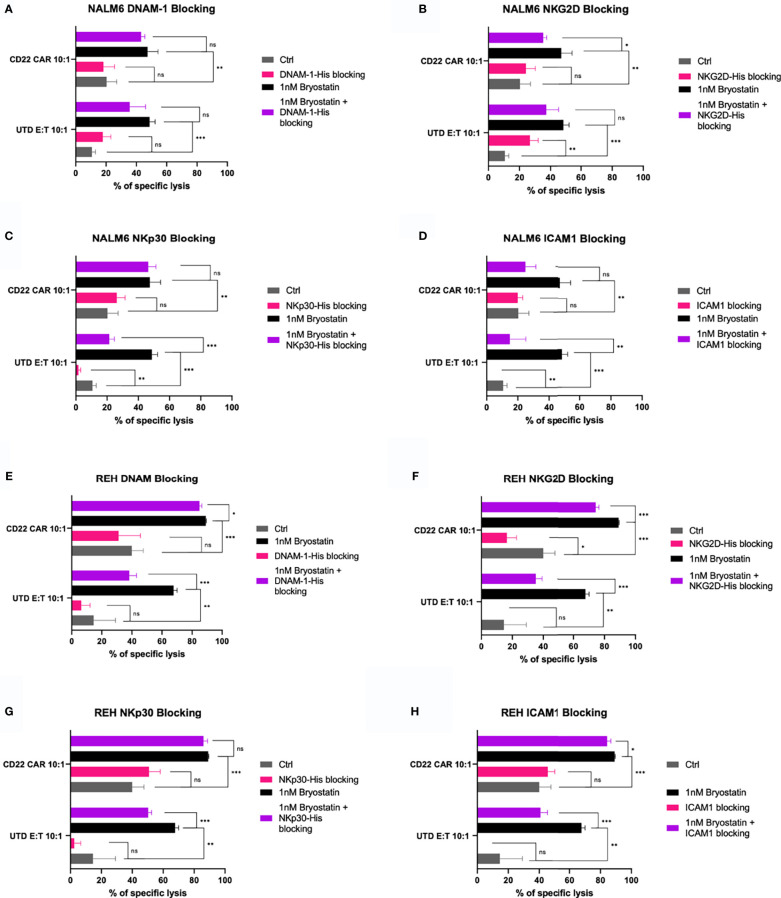
Blocking of innate immunoreceptor ligands during CAR-T mediated cytolysis. The NALM6 pre-B ALL cell line was exposed to anti-CD22 CAR-T or control UTD cells at an E:T ratio of 10:1, y-axis. Results are grouped in each panel by cytolysis seen with untreated target (gray, control), bryostatin treatment (black), or treated with blocking agent (pink) or blocking agent and bryostatin (purple), using **(A)** recombinant DNAM-1, **(B)** NKG2D, **(C)** NKp30, or **(D)** anti-ICAM1 antibody, for 30 minutes prior to addition of effector cells. Average of 3 replicate wells are shown, with statistical difference between groups plotted, ns p > 0.05, *p < 0.05, **p < 0.01, ***p < 0.001. **(E–H)** REH leukemia cells were similarly analyzed.

To assess the contribution of known NK ligands on bryostatin-induced cytolysis of leukemia targets, we used both antibody and soluble protein-based inhibition assays. When NALM6 cells with or without bryostatin treatment were used as CD22 CAR-T or UTD targets, we again saw significant induction of UTD-mediated leukemia cell cytolysis induced by bryostatin treatment, **Figures 11A–D**. The addition of soluble DNAM-1 did not have an effect. NKG2D and ICAM did have some effect on bryostatin-induced killing by CD22 CAR-T. UTD was most affected by NKp30 and ICAM-1 blocking. Likewise, NKG2D and ICAM1 blocking impacts killing of bryostatin-treated REH by CD22 CAR-T and UTD, **Figures 11F, H**. NKp30 effects were limited to UTD for REH, just as for NALM6, **Figure 11G**. REH differed to a degree in that DNAM1-blocking now was shown to have an effect, and to a greater degree for UTD upon bryostatin treatment, **Figure 11E**.

**Figure 12:**
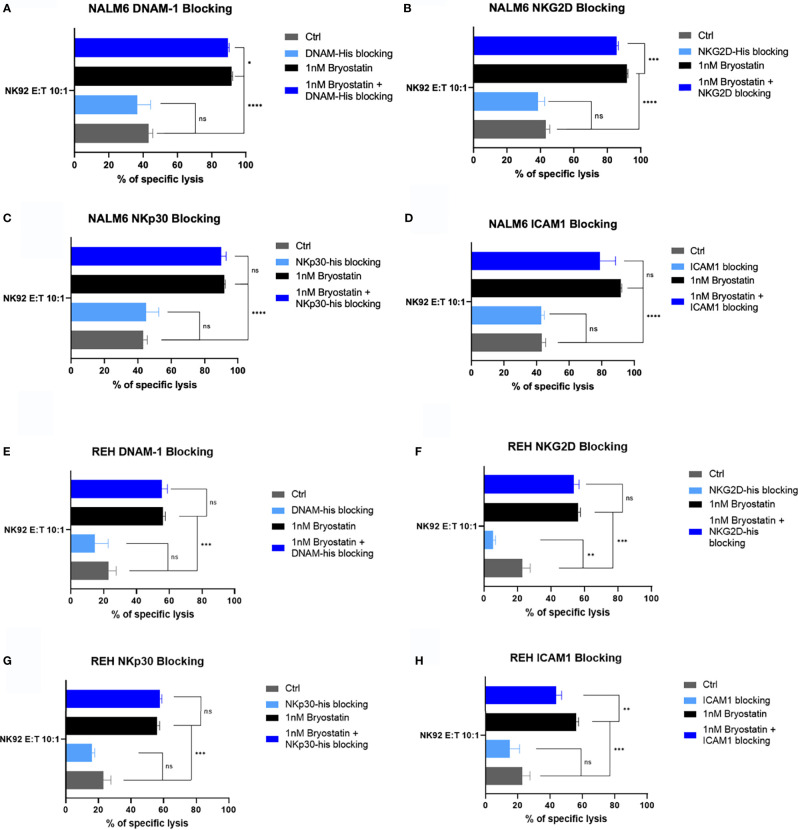
Blocking of innate immunoreceptor ligands during NK92-mediated cytolysis. The NALM6 pre-B ALL cell line, with or without bryostatin-treatment, was cultured with NK92 cells at an E:T ratio of 10:1. Treatment groups are arranged according to the blocking agent tested: **(A)** DNAM-1, **(B)** NKG2D, **(C)** NKp30, or **(D)** anti-ICAM1 antibody as in **Figure 11**. Average of 3 replicate wells are shown, with statistical difference between groups plotted, ns p > 0.05, *p < 0.05, **p < 0.01, ***p < 0.001, ****p < 0.0001. **(E–H)** REH leukemia cells were similarly analyzed.

Because Raji cells are universally sensitive to NK92 mediated killing, we restricted our analysis of NK92-mediated killing to the pre-B ALL lines. NALM6 killing was not impacted to a great degree by any of the 4 blocking agents tested. Although NKG2D blocking gave a statistically significant effect, the overall effect was small, **Figure 12B**. ICAM1-blocking did inhibit REH killing in the presence of bryostatin. NKG2D blockade had an effect on non-treated REH, but this difference was lost when bryostatin was added, as the overall killing was increased, **Figure 12F**. Taken together, we can assert that the decrease of ICAM1-mediated cell adhesion impacted bryostatin-induced killing by all three effectors tested, but impacted NK92-mediated killing less. NKG2D blockade impacted T cell mediated killing (both CAR-T and UTD), while NKp30 had activity in UTD but not CAR-T cell-mediated killing. Our findings indicate that well-characterized mediators of NK-like killing did have an effect in our system. However, the killing mechanisms are complex, and likely additive as no single blocking agents inhibited all killing activity. We can also conclude that the multifactorial nature of innate immune cell-mediated cytotoxicity is activated in a novel way by the addition of bryostatin, as demonstrated herein.

### Potential Bryostatin Epigenetic Changes Impacting Leukemia Cell Target Expression

We also carried out a comprehensive read analysis of Cut&Tag data, comparing bryostatin-treated and untreated REH, NALM6 and Raji cells (**Supplementary Figure S8**). Raji changes were the most dynamic, and NALM6 showed very few significant changes (The file comprising **Supplementary Table 4** contains the complete data set). We also specifically inspected the ligands for innate immune receptors that were expressed or induced in effector cells as detected by flow cytometry, **Supplementary Table 3A**. Among those ligands, HLA-ABC which would interact with iKIRs, changed the most, **Supplementary Table 3B**. We did very little to explore the Fas system in functional assays due in part due to the unchanging expression of FasL on effector cells, and low expression of Fas on NALM6 and REH. Although expressed on Raji cells, bryostatin did not alter Fas expression.

Promoter regions for ligands known to be important in NK cell activity were also compared by Cut&Tag analysis. Activating ligands (MICA, MICB, ULBP1, ULBP2, ULBP3, Nectin-2 (CD112), PVR (CD155); and inhibitory ligands (HLA-E, Nectin-1/CD111), showed no large alterations. NKp30 and NKp80 ligands (B7H6, BAG6, and CLEC2B) also were unchanged. Fas, TRAILR1 (DR4) and TRAILR2 (DR5) were also unchanged. Analysis of the SLAM family (FLAMF1, SLAMF3/LY9, SLAMF4/CD244, SLAMF4/CD84. SLAMF6, SLAMF7, SLAMF4LG/CD48) also showed no bryostatin effects. Analysis of KIR ligands HLA-A,-B, and -C showed no changes. HLA-G also was not changed, showing only H3K27me3 (inhibitory mark) reads present. Thus, we documented the presence and activity of well described innate immune receptors active in our system. Any single change in expression, as detected by flow cytometry, or attempts in direct protein blockade in functional assays reveal that these signals are integrated from multiple inputs. Further work remains to explain the specific signals operative in any one effector cell or cell line. The effects of bryostatin are layered on to the biology of CD19 and CD22 as expressed by B-ALL cell lines. The upregulation of these molecules does sensitize ALL to cytolysis, but one must include in the analysis of CAR-T activity the strong induction of NK-like and CTAK killing activity, above and beyond CD22 antigen upregulation. Furthermore, bryostatin cannot be assumed to be universally applicable to B cell malignancies as Raji cells are rendered insensitive to cytolysis upon treatment.

## Discussion

B cell activation is a carefully regulated event. In addition to antigen- or developmentally-initiated positive signals, regulatory or inhibitory signals, like those mediated by CD22, are required to prevent hyperactivation (40). In keeping with the diversity of activity of the Siglec (sialic acid binding Ig-type lectin) family of receptors, CD22 has both negative regulatory activity, mediated through intracellular ITIM motifs that recruit SHP-1 and Grb2, as well as endocytic activity for ligands bearing specific glycoform structures, notably alpha2,6-linked sialic acid (41, 42). Recent studies with the B cell line DT40 have demonstrated that CD22 internalizes into early endosomes *via* clathrin-mediated endocytosis following B cell receptor (BCR) stimulation (43). Upon internalization, CD22 can either be marked for degradation by the E3 ubiquitin ligase cullin 3, or circulate back to the cell surface membrane, revealing a complex network amenable to multiple regulatory inputs. Thus, a number of clinically-relevant epigenetic modifiers or differentiation agents were explored, with bryostatin showing the broadest impact across the 3 lines tested on CD22 surface expression.

The average site density of CD22 on clinical pediatric ALL samples is 3,470 with a broad range (349-19,653) that is dependent in part on disease subtype (44). In an effort to overcome the evasion of B-ALL from CAR-T therapy, Ramakrishna et al., demonstrated that bryostatin is able to upregulate CD22, and to improve outcomes in a NSG animal model system (13). Laboratory and clinical studies have revealed that very little CD22 is shed, and although a possibility in our system, the evasion of immune effector cells by increased antigen target shedding is unlikely (45). The internalization of CD19 and CD22 was carefully described in studies evaluating anti-CD22 and anti-CD19 immunotoxins. In these studies, CD19 was expressed at 3-4 fold higher with respect to site density, but was far less effective as an anti-leukemic target for antibody-linked toxins due to its lower rate of internalization (46). Thus, there is documented differential internalization rate, even though the number of CD19 on the surface of B cell lines always exceeds that of CD22 (46). Immunofluoresence studies revealed that antibody-mediated ligation drives these receptors into the same intracellular compartment. This indicates both a differential mechanism with regard to ligation-dependent internalization, and some commonality as the initial endosomal compartment is the same. A global coregulation of CD19 and CD22 is also suggested by the lower levels of CD22 on ALL relapse post CAR-19 therapy (47). We explored the activity of a number of epigenetic modifiers and differentiation agents, **Figure 1**, to determine if other clinically relevant agents modulate CD22 target number on the cell surface, apart from overt cytotoxic activity.

Although panobinostat and vorinostat may stabilize antigen expression, only bryostatin appeared to consistently upregulate target antigen expression, and thus we continued our studies by focusing on bryostatin. Bryostatins are a family of cyclic polyketides, with most research focused on bryostatin 1 (48). The activity of bryostatin 1 is attributed to its interaction with the diacylglycerol biding site of the C-1 regulatory domain of protein kinase C. Upregulation of CD22 was noted alongside an increase in cell size and membrane projections in bryostatin-treated CLL (chronic lymphocytic leukemia) cells. Importantly, the effects of bryostatin on PKCbII change from activating to inhibitory with increased dosage or time in culture (14). At the lower concentration of 1 ng/mL, bryostatin induces CLL differentiation activating both PKCbII and Erk (49). Thus, it has a dual concentration-dependent effect. To examine the epigenetic effects of bryostatin we employed Cut&Tag analysis of two histone modifications associated with promoting transcription, H3K4me2 and H4K4me3, and one modification associated with repressing transcription, H3K27me3. CD19 and CD22 were not overtly altered, **Supplementary Figures S2, S3**, in keeping with the relatively unaltered overall transcript and protein levels, **Figure 2**. Responsiveness to bryostatin was clearly an attribute of transformed B cells, as normal B cell surface expression of both CD22 and CD19 was unaltered by bryostatin, **Figure 1**. The modulation of both CD19 and CD22 may be key attributes of successful CAR-T therapy, and will be explored in future studies. The ability of a CAR-T cells to release from a specific target and engage in serial killing would be inhibited if surface expression of the target molecule remained unchanged.

In addition to bryostatin treatment, we sought to determine the effect of CAR-T cells on cell surface CD22 expression. Much to our surprise, anti-CD22 CAR-T down-regulated both CD22 and the off-target antigen CD19, **Figure 3**. Increasing E:T ratios resulted in a greater decrease in CD22 and CD19 surface expression. This data suggests that the addition of bryostatin may keep target antigen surface expression higher and allow for a greater degree of CAR-mediated leukemia cell killing. This raises a key question, are we are selecting for a low antigen-expressing leukemia sub-clones, or observing antigen recycling and internalization at the cellular level? When the CAR-T + bryostatin challenged leukemia cells were isolated and re-cultured separately, interesting long-term changes were observed, that resolved in a week for 2 of the 3 lines, **Figure 4**, indicating that clonal selection was not the operative mechanism for detecting an antigen low population. For the Raji cell line, on-target CD22 expression decreased due to the addition of CAR-T. This effect lasted throughout 72 hours of post CAR-T co-culture, but normalized by day 7. For CD19 modulation in Raji, CD22 CAR-T induced CD19 down-modulation irrespective of bryostatin treatment. Effects on NALM6 CD19 and CD22 surface expression were not as dramatic, and returned to original levels by day 7. CD22 down-modulation by CAR-T was essentially reversed by bryostatin within 24 hours, indicating a long-term dominant effect that resolved within a week. The results seen with REH were unexpected in that there was a strong rebound effect for cultures treated with anti-CD22 CAR-T and bryostatin. While CAR-T only and bryostatin-only cultures normalized CD22 expression levels by day 7, the combined treatment invoked a more permanent change in that even on day 7, CD22 and CD19 surface expression levels remained high. The genetic or epigenetic basis for this change will be explored in future studies. Our data illustrates that bryostatin has a profound effect on target antigen expression, even days after it is removed from the culture media.

Another potential mechanism for the loss of antigen expression on the target cell is trogocytosis mediated by the CAR-T cell. Antigen acquisition by CAR-T cells was detectable, and mirrored the relative antigen expression on each leukemia target cell, **Figure 5**. Importantly, this was an antigen non-specific process. The level of trogocytosis was also partially reflective of the degree of leukemia cell killing. Less transfer was seen was seen with higher E:T ratios. This may be due either to the greater number of T cells that can receive membrane associated surface antigens (signal dilution), or that cells being actively lysed do not “donate” membrane and membrane-associated proteins.

Investigating the cytolysis of bryostatin-treated leukemia cell lines gave unanticipated findings, **Figure 6**. Untreated Raji cells were readily killed by CD22 CAR-T. However, when bryostatin was added, killing was completely abrogated. Untransduced T cells (UTD) are activated T cells treated exactly like anti-CD22 CAR-T, with the exception that no LV vector transduction takes place. Both REH and NALM6 were efficiently killed by CAR-T, while UTD showed a very low killing activity, as expected. However, when bryostatin was added, UTD now mediated strong REH and NALM6 killing. This may indicate that the increased killing of REH and NALM6 is not due to the increased number of CD22 molecules on the leukemia cell surface, but due to bryostatin-induced innate immune ligands that make the cells susceptible to CAR-T and UTD antigen non-specific killing. Neither CAR-T nor UTD lysed K562 cells, indicating that the increased B cell leukemia killing was not mediated by standard NK cell interactions. Cold-target inhibition demonstrated that the bryostatin-induced killing of ALL lines could be blocked by the innate immune ligands expressed by K562, while preserving CAR-T mediated killing.

To specifically explore leukemia cell line sensitivity to NK cell killing, we used the NK92 cell line, **Figure 7**. Unlike CAR-T or UTD, NK92 had strong cytolytic activity against K562 cells. And, as expected this was abrogated with K562-mediated cold target inhibition. Raji cells were very sensitive to NK92 killing, with or without bryostatin addition, and this killing was completely unaffected by cold-target inhibition. NALM6 and REH were not killed by NK92 unless they were first treated with bryostatin. This killing also was not abrogated by K562-mediated cold target inhibition. These results indicate that CAR-T cells mediate killing through a number of mechanisms that include the CAR itself, NK-like killing that can be blocked by K562-mediated cold target inhibition, and killing induced by ligands induced by bryostatin. This led us to propose a new model for CAR-T mediated killing of bryostatin-treated cells, **Figure 8**. We now use the term CTAK (CAR-T activated killing) to refer to off-target cytotoxicity against B-ALL cell lines mediated by CAR-T cells. Moreover, CTAK activity is optimized by bryostatin treatment of target ALL cell lines.

The striking evasion of bryostatin-treated Raji cells to CAR-T cell-mediated cytotoxicity, but not NK92, led us to hypothesize that activation of EBV latency may be responsible for immune evasion. While the EBV genome present in Raji cells is not replication competent, and thus gangiclovir effects may not be directly EBV-related, the latent EBV genome present in Raji cells remains a focus of study on the immune evasion mechanisms utilized by EBV (50, 51). Preliminary RNASeq studies of bryostatin-treated Raji highlighted EBV reactivation pathways (not shown). When we treated Raji with ganciclovir, some sensitivity to CAR-T-mediated cytolysis was recovered, **Figure 9**. This did not correlate with epigenetic changes in control regions for EBNA-1 or EBNA-2/LP expression, nor were changes seen in the promoter regions associated with EBV reactivation from latency, Zta, Rta, and LF1,2,3 (**Supplementary Figure S6**). Upon examining other EBV latency promoters we noticed a marked increase in reads for BLLF1. BLLF1 encodes the major viral envelope glycoprotein gp350. Although gp350 does interact with B cell surface proteins, notably CR2/CD21, we did not explore this finding further in this report. Due to the minor role EBV latency gene expression plays in bryostatin-treated Raji immune evasion, and the examination of only one EBV-positive line, we cannot make a causal link to immunoevasion and EBV. An alternate hypothesis would be the effect bryostatin has on α2,6 sialic acid-bearing targets, which if increased would impact CD22 expression.

In our final set of studies we explored the contribution of ligands known to be involved in innate immune recognition of cancer targets. For NALM6, ICAM-1 blockade diminished CAR-T mediated cytolysis, **Figure 11D**. There was a decrease when NKG2D was blocked as well, but this difference did not reach statistical significance. DNAM1 blockade did very little in any of our assays, in opposition to previous reports showing DNAM-1 activation of NK cells *via* interaction with CD112 (Nectin-2) and CD155 (PVR) on myeloid leukemias (52). The REH cell line showed decreased CAR-T and UTD cytolysis when either ICAM-1 or NKG2D were blocked, **Figure 11F, H**. NK92 cell-mediated killing of NALM6 and REH was impacted by NKG2D or ICAM1 blockade, **Figure 12**. The only evidence of NKp30 activity in our assays was the partial blockade of UTD-mediated killing of bryostatin-treated REH or NALM6, **Figures 11C, G**. Due to the low expression of the NKp30 ligands B7H6 and BAG6 on leukemia target cell lines, we hesitate to ascribe this activity as being a key point of differentiation between the killing activities we described, but it indicates that transduction with a CAR may give rise to a different innate immune effector activity than that seen in UTD. In sum, the receptors tested as a single agents had a moderate effect. This implies that the killing activities observed are the result of additive signals that are integrated by the effector cell type being tested.

**Figure 13:**
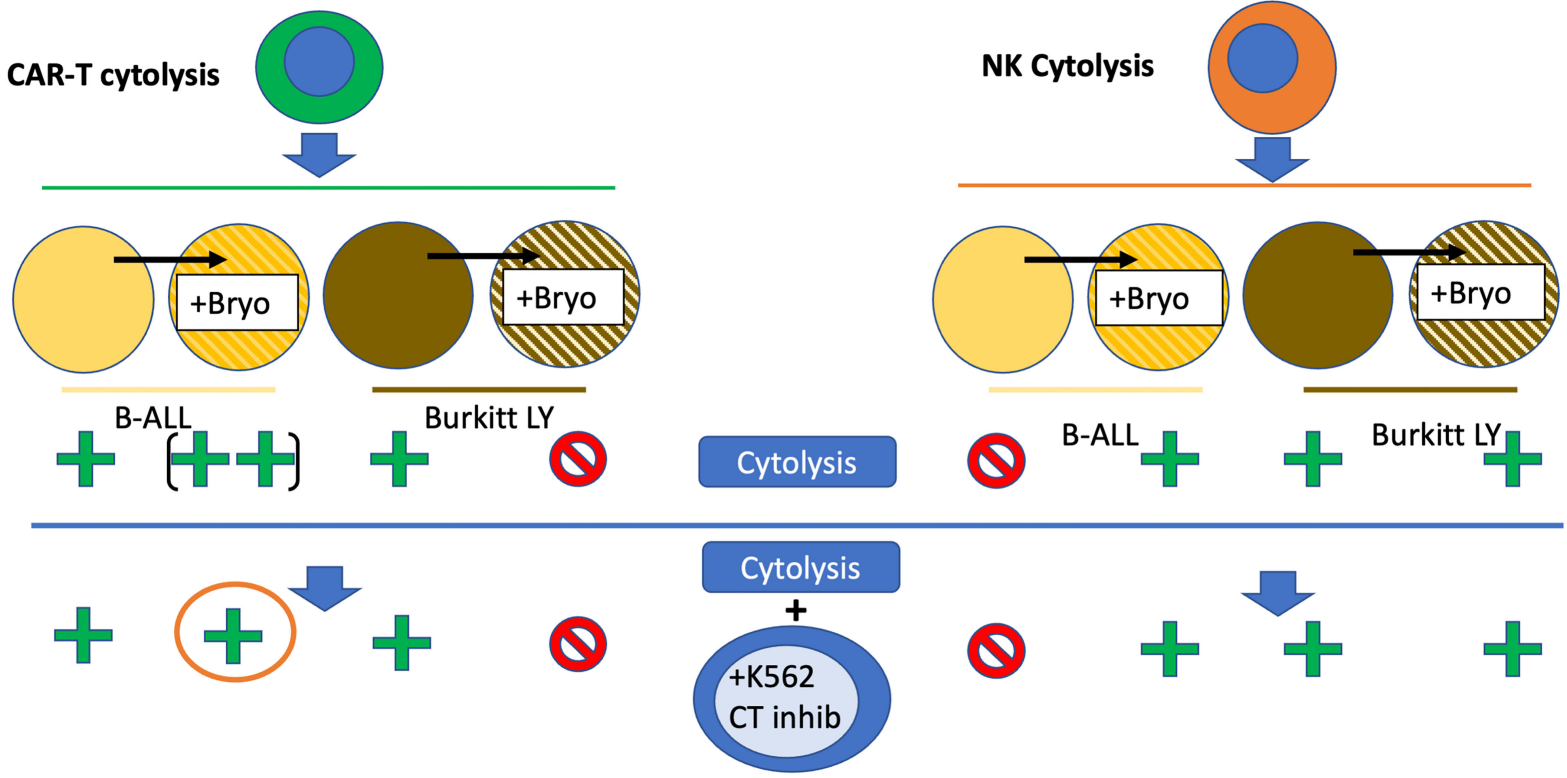
Bryostatin modulation of CAR-T and NK92-based leukemia cell line cytolysis. Bryostatin treatment of pre-B ALL (yellow to striped yellow) and Burkitt (brown to striped brown) cell lines alters sensitivity to effector cell cytolysis. CAR-T cytolysis (left half, green cell) is amplified by bryostatin treatment (+ to ++) for B-ALL, and blocked for Burkitt’s (+ to -). Progressing to the lower portion of the figure illustrates the effect of K562 cell mediated cold-target inhibition (+K562 CT inhib). CAR-T mediated killing of Burkitt’s is unaffected, while some of the bryostatin facilitated killing of B-ALL is lost (orange circle). For NK cytolysis (orange cell, right half), the induction of a new set of innate immune ligands that now allow for killing of B-ALL is illustrated (- to +). Burkitt’s remains unaffected, and universally sensitive. The induced ligands on B-ALL are unaffected by cold-target inhibition and remain sensitive to NK cytolysis.

Bryostatin profoundly modulates cell surface antigen expression of targeted leukemia cells. For NK92-mediated killing, bryostatin induced a set of ligands on the B-ALL cell lines REH and NALM6 that allowed them to be recognized and eliminated. Moreover, these signals were not those normally associated with NK cell activity, as cold target inhibition had no effect, **Figure 13**. For the more developmentally mature B cell line, Raji Burkitt lymphoma, bryostatin had no effect. However, Raji cells are universally sensitive to NK92. Effects on T cell-mediated killing of Raji were very different from NALM6 and REH. T cell killing of Raji was completely abrogated by bryostatin. This surprising result indicates that bryostatin cannot be assumed to be universally beneficial in CD22 CAR-T mediated killing. This also indicates that the increased killing cannot be solely attributed to an increase in the number of CD22 molecules on the cell surface. A portion of the bryostatin-amplified susceptibility to cytolysis is blocked by cold-target inhibition, indicating that canonical NK receptor interactions play a role for ALL. We have termed the non-canonical activity that could not be blocked by cold-target inhibition CTAK (CAR-T cell antigen non-specific killing) in order to differentiate it from NK cell and LAK cell-mediated killing.

Analysis of cell surface antigen dynamics revealed that trogocytosis occurs to some degree, but is unlikely to be a major source of antigenic modulation seen during CAR-T cell mediated killing. The dual activity of CAR-T and bryostatin induced changes in surface antigen expression for days, even when these agents were removed, **Figure 4**. Notably, the REH cell line maintained changes in both CD22 and CD19 antigen expression levels for 7 days when co-cultured with both CAR-T and bryostatin. Epigenetic analysis at 24 hours revealed changes in EBV antigen expression control regions in Raji cells, and alterations for other proteins as well, but not in the control regions of CD19 and CD22 (**Supplementary Table 2**). The complex dynamics of surface antigen expression in leukemia cells will be the focus of future studies. A recent analysis of CD22 CAR-T treated patients revealed that in addition to T cell exhaustion and a lack of stimulation due to antigen down-modulation, significant splice variations in CD22 have also been noted that may account for escape from immunotherapeutic control (53).

We have demonstrated that bryostatin induces innate immune receptor ligands on ALL that increase CAR-T cell killing, which can be blocked only in part by cold-target inhibition with K562. We have also demonstrated that Raji cells are rendered resistant to T cell mediated, but not NK92-mediated killing, by bryostatin. Furthermore, NK92 targets are induced on B-ALL when treated with bryostatin, and these also are not influenced by cold-target inhibition. We have described the mechanisms behind these effects only in part. Anti-ICAM1 antibody seems to partially block these effects for both T and NK effector cell types, and other innate immune receptors clearly play a role as well. We propose that for clinical studies where CAR-T cells are combined with bryostatin, that the leukemia cell type targeted should first be documented to have increased biological sensitivity to cytolysis. A simple increase in CD22 target cell number is not sufficient. Secondly, the addition of NK cells to CAR-T cell therapeutic approaches may overcome escape mechanisms that more mature leukemia subtypes display, and should be considered on their own or in combination with bryostatin.

## Data Availability Statement

The datasets presented in this study can be found in online repositories. The names of the repository/repositories and accession number(s) can be found below: https://www.ncbi.nlm.nih.gov/geo/query/acc.cgi?acc=GSE192965 and https://www.ncbi.nlm.nih.gov/geo/query/acc.cgi?acc=GSE192837.

## Author Contributions

The studies were conceived and designed by AL and RO. Experiments were carried out by LW, EA, and YZ. Bioinformatics analysis was carried out by YZ. Studies were supervised by RO. All authors contributed to the article and approved the submitted version.

## Funding

This study was supported by the Seattle Children’s Foundation, Seattle Children’s Research Institute and the Seattle Children’s CBDC Research Pilot Funds Program.

## Conflict of Interest

RO has received research support from Miltneyi Biotec unrelated to this work. He has also consulted for Umoja Biopharma and Abound Bio, neither of which is related to this work.

The remaining authors declare that the research was conducted in the absence of any commercial or financial relationships that could be construed as a potential conflict of interest.

## Publisher’s Note

All claims expressed in this article are solely those of the authors and do not necessarily represent those of their affiliated organizations, or those of the publisher, the editors and the reviewers. Any product that may be evaluated in this article, or claim that may be made by its manufacturer, is not guaranteed or endorsed by the publisher.

## Supplementary Material

Supplementary Figure 1 (S1) Heatmap of histone enrichment anchored at TSS (Transcription Start Sites). Raji, NALM6, and REH cell lines were treated with bryostatin for 24 hours (Bryo), and then analyzed by Cut&Tag, or left untreated, Ctrl. Total reads were normalized by RPKM, aligned for transcriptional start sites, and signals for H3K4me2, K3K4me3, and H3K27me3 compared to untreated cells cultured in parallel. Both read length from the transcriptional start site (bottom scale), and frequency (vertical scale) are indicated.

Supplementary Figures 2 and 3 (S2 and S3). Cut&Tag analysis of CD22 and CD19 transcriptional control regions. Cut&Tag reads mapped to genome regions encoding S2) CD22 and S3) CD19, are displayed for Raji, NALM6, and REH cell lines as listed on the y-axis. Next to each cell line the three immunoprecipitating antibody specificities are listed along with the IgG control antibody (H3K4me2, H3K4me3, H3K27me3, IgG). For each condition, two read tracks are presented for cell lines that have been treated (+Bryostatin) or untreated control samples. Thus, changes due to bryostatin treatment appear directly below the untreated cell line sample. Below each data set the corresponding genome map is illustrated. To the right of each panel, average of normalized total reads within the gene region from 3 experiments are presented for H3K4me2 and K3K4me3. For CD22: Raji showed increased H3K4me2 and H3K4me3 (p<0.001), REH (p<0.001) and NALM6 (p<0.05) showed increased H3K4me3 reads. n.s., not significant. For CD19 only H3K4me3 (p<0.05) was increased by bryostatin treatement.

Supplementary Figures 4 and 5 (S4 and S5). Modulation of the EBV genome in Raji leukemia cells mediated by bryostatin. S4) Sequence reads for two of the major transcriptional activators of EBV latency, EBNA-1 and EBNA-2/LP analyzed by Cut&Tag analysis and mapped on the EBV genome for the presence of epigenetic modification of H3K4me2, H3K4me2, and H3K27me3 (y-axis). No reads were detected for the IgG control. Reads are presented as parallel samples for bryostatin-treated (+bryostatin) or untreated Raji. Shown below the immunoprecipitated Cut&Tag reads are total mRNA reads (in gray) displayed over the relevant portion of the EBV viral genome, shown at the bottom portion of the plot. S5) Analysis of the viral genome encoding two of the latency antigens that mediate B cell activation, C) LMP1/2 and D) LMP2A/B, demonstrated no overt changes in Cut&Tag signal, and a decrease in total mRNA reads for LMP-1 and LMP-2.

Supplementary Figure 6 (S6) Cut&Tag analysis of EBV latency reactivation promoters in Raji. Cut&Tag Profiles for **(A)** Zta/Rta, **(B)** BLLF1, and **(C)** LF1,2,3 are presented for reads amplified from H3K4me3, H3K4me3, K3K27me3 immunoprecipitations, as in S2. Reads are presented in parallel with or without (+bryostatin) bryostatin treatment. Red square indicates difference in H3K4me3 reads for BLLF1.

Supplementary Figure 7 (S7) Flow cytometry for innate immune ligands: effector cell and leukemia cell line characterization. **(A–N)** the three immune effector lines (NK92, UTD, and CD22 CAR-T) were characterized for expression of NKG2D, DNAM-1, NKp30, NKp44, NKp46, TRAIL, FasL, KIR2DL1/DS1, KIR3DL1/DS3, NKG2A, ICAM1, ICAM2, LFA1, and CD56. All antibodies used are listed in Methods. **(O-BB)** Four leukemia lines (NALM6, REH, Raji, K562) were analyzed for expression of MIC-A/B, ULBP1, ULBBP-2/5/6, ULBP3, Nectin-2, B7H6, BAG6, DR4, DR5, Fas, HLA-A,B,C, HLA-E, ICAM1, ICAM2. In all flow panels isotype control (gray), untreated cells (blue), and bryostatin-treated cells (red, 1 nM overnight) are compared.

Supplementary Figure 8 (S8) Volcano plot of differentially enriched peak by Cut&Tag analysis. Enriched peak reads were normalized by read depth and compared between samples (control versus bryostatin treatment) by DESeq2. Shown is H3K4me2 (top row) and H3K4me3 signal (bottom row) for REH **(A, D)** NALM6 **(B, E)**, and Raji **(C, F)**. Green color indicates gene expression differential of >4-fold change and p<0.001 in H3K4me2, gene expression differential of >4-fold change and p<0.005 in H3K4me3. Gene identities can be found in ST2.

Supplementary Table 1 (ST1) Epigenetic modifiers and differentiation agents modulate CD19 and CD22 expression at non-cytotoxic concentrations. Three cell lines (Raji, NALM-6, REH) were tested across a broad range of concentration to ensure a noncytotoxic concentration of epigenetic modifiers and differentiation agents were tested for the ability to increase the expression of the number of CD22 and CD19 molecules per cell, **Figure 1**. Agents that changed the expression of each target antigen are indicated by the greatest percent increase of the number of CD22 or CD19 molecules per cell are listed.

Supplementary Table 2 (ST2) Expression of innate immune receptors on effector cells. MFI signal, (summarized from Supplementary Figure 7) for NK92, untransduced T cells (UTD T) and CD22 CAR-T cells, for expression of surface innate immune effector molecules. NK92 expression patterns are consistent with primary NK cells, while UTD and CAR-T cells lack CD56, NKp30, and NKp44 expression. Bryostatin has negligible effects on NK-92, and some induced (ind) expression of TRAIL and ICAM-1 on UTD and CAR-T cells.

Supplementary Table 3 A, 3B Summary of gene expression by MFI, based on flow cytometry profiles, S3. **Table 3A**) Effector cells (NK92, UTD, CD22 CAR-T) with or without bryostatin treatment (+Bryo) were analyzed for expression of Activating Ligands (orange blocks, column 1): MFI for NKG2D, DNAM1, NKp30, NKp44, NKp46, TRAIL, FasL; and for Inhibitory Ligands (blue blocks, column 1) KIR2DL1/DS1, KIR3DL1/DS1, NKG2A; and for adhesion receptors (gray blocks, column 1) ICAM1, ICAM2, LFA1, CD56 are shown. **Table 3B)** NK ligands (column 3) interacting with Activating Receptors (column 2, orange blocks), Inhibitory Receptors (column 2, blue blocks), and adhesion receptor (gray block, column 1) are listed according to expression on leukemia cell line targets (NALM6, REH, Raji, K562) that have been untreated or treated (+bryo) with bryostatin. MFI for MIC-A/B, ULBP1, ULBP-2/5/6, ULNP3, NECTIN2, B7H6, BAG6, DR4, DR5, Fas, HLA-A,B,C, HLA-E, ICAM1, and ICAM2 are shown. MFI was calculated by (geometric mean of ligand fluorescence – geometric mean of isotype fluorescence).

Supplementary Table 4 (ST4) Total reads and reads exceeding threshold. Table (ST2_CutNTag_differential) describes methylation patterns (me2 and me3) of histone 3 (H3) modifications at lysine 4 (K4). The first of each pair of tabs reports all signal from Cut&Tag analysis and the second tab lists differential expression between bryostatin-treated and control (non-treated) leukemia cells. The global tab contains the peak ID (column A), chromosome (column B), start and end sequence number (C,D), annotation (E), distance to transcription start site (TSS) (F), gene name (G), gene description and type (H,I). Signal for the cell lines, in triplicate, analyzed without bryostatin (J-L) with bryostatin (M-O), normalized signal by DESeq2 without bryostatin (P-R), normalized signal by DESeq2 with bryostatin (S-U); column V is not utilized, but gives signal intensity mean across all samples, column W, log2 fold-change (without bryostatin versus with bryostatin, and thus negative in value); column X, standard error of log2 fold-change; column Y, standard error of the log2 fold-change, Wald statistic; column Z, Wald test p-value; column AA, Benjamini-Hochberg adjusted p-value. All stats were calculated within the DSeq2 package. Thus, ST2 contains the following tabs: K4me2NALM6 K4me3NALM6_|FC|>4_P<0.001_green (fold change greater than 4, indicated p value) K4me3NALM6 K4me3NALM6_|FC|>4_P<0.005_green K4me2REH K4me2REH_|FC|>4_P<0.001_green K4me3REH K4me3REH_|FC|>4_P<0.005_green K4me2Raji K4me2Raji_|FC|>4_P<0.001_green K4me3Raji K4me3Raji_|FC|>4_P<0.005_green.
