# Supplementary material for: Bryostatin Activates CAR T-Cell Antigen-Non-Specific Killing (CTAK), and CAR-T NK-Like Killing for Pre-B ALL, While Blocking Cytolysis of a Burkitt Lymphoma Cell Line
Source: Front Immunol. 2022 Feb 9;13:825364. doi: 10.3389/fimmu.2022.825364 (PMC8864095; doi:10.3389/fimmu.2022.825364)
Supplement: Supplementary Figure 1 — (S1) Heatmap of histone enrichment anchored at TSS (Transcription Start Sites). Raji, NALM6, and REH cell lines were treated with bryostatin for 24 hours (Bryo), and then analyzed by Cut&Tag, or left untreated, Ctrl. Total reads were normalized by RPKM, aligned for transcriptional start sites, and signals for H3K4me2, K3K4me3, and H3K27me3 compared to untreated cells cultured in parallel. Both read length from the transcriptional start site (bottom scale), and frequency (vertical scale) are indicated. [file Presentation_1.ppt]

## Slide 1
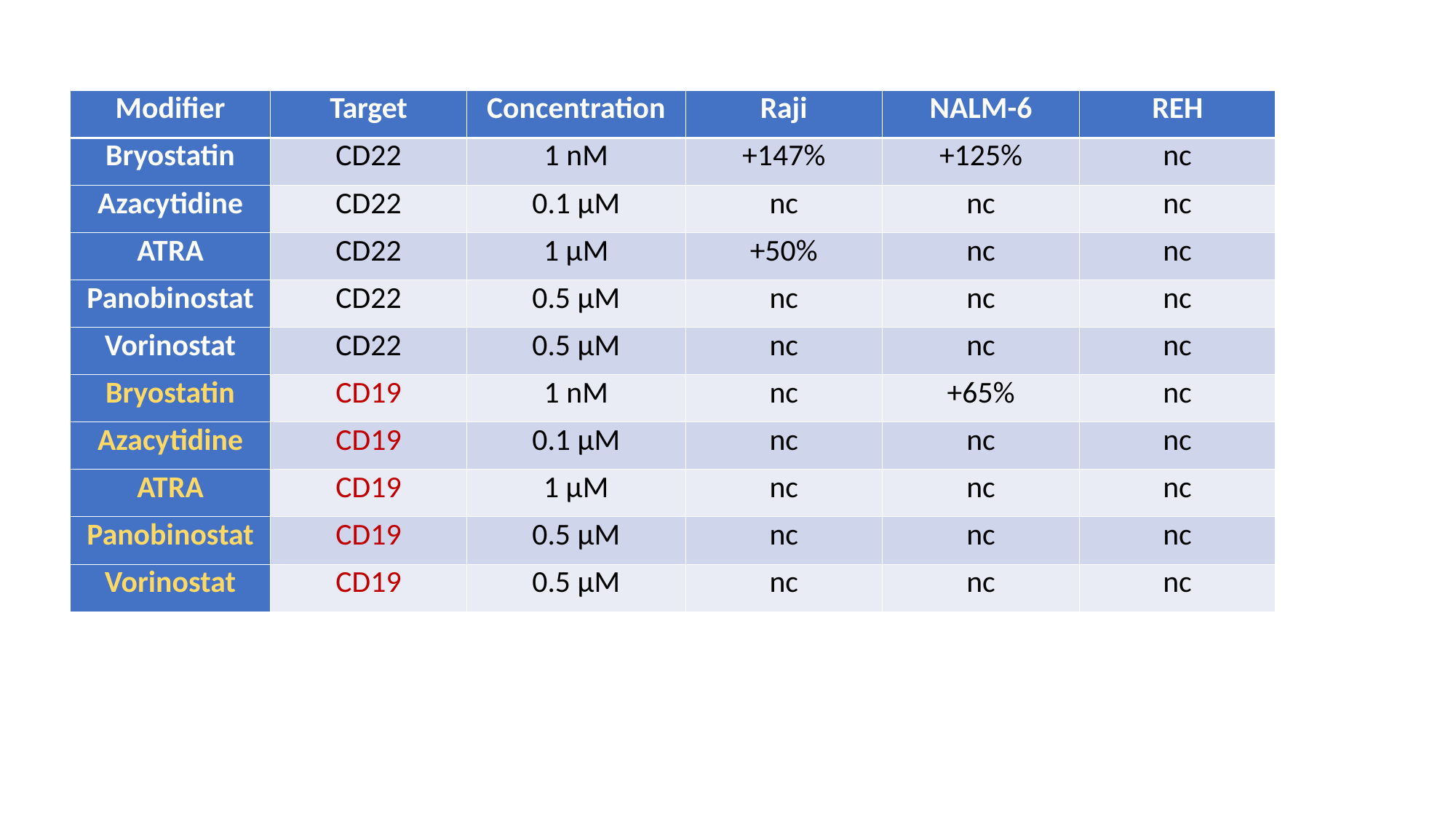

| Modifier | Target | Concentration | Raji | NALM-6 | REH |
| --- | --- | --- | --- | --- | --- |
| Bryostatin | CD22 | 1 nM | +147% | +125% | nc |
| Azacytidine | CD22 | 0.1 µM | nc | nc | nc |
| ATRA | CD22 | 1 µM | +50% | nc | nc |
| Panobinostat | CD22 | 0.5 µM | nc | nc | nc |
| Vorinostat | CD22 | 0.5 µM | nc | nc | nc |
| Bryostatin | CD19 | 1 nM | nc | +65% | nc |
| Azacytidine | CD19 | 0.1 µM | nc | nc | nc |
| ATRA | CD19 | 1 µM | nc | nc | nc |
| Panobinostat | CD19 | 0.5 µM | nc | nc | nc |
| Vorinostat | CD19 | 0.5 µM | nc | nc | nc |
Table S1

## Slide 2
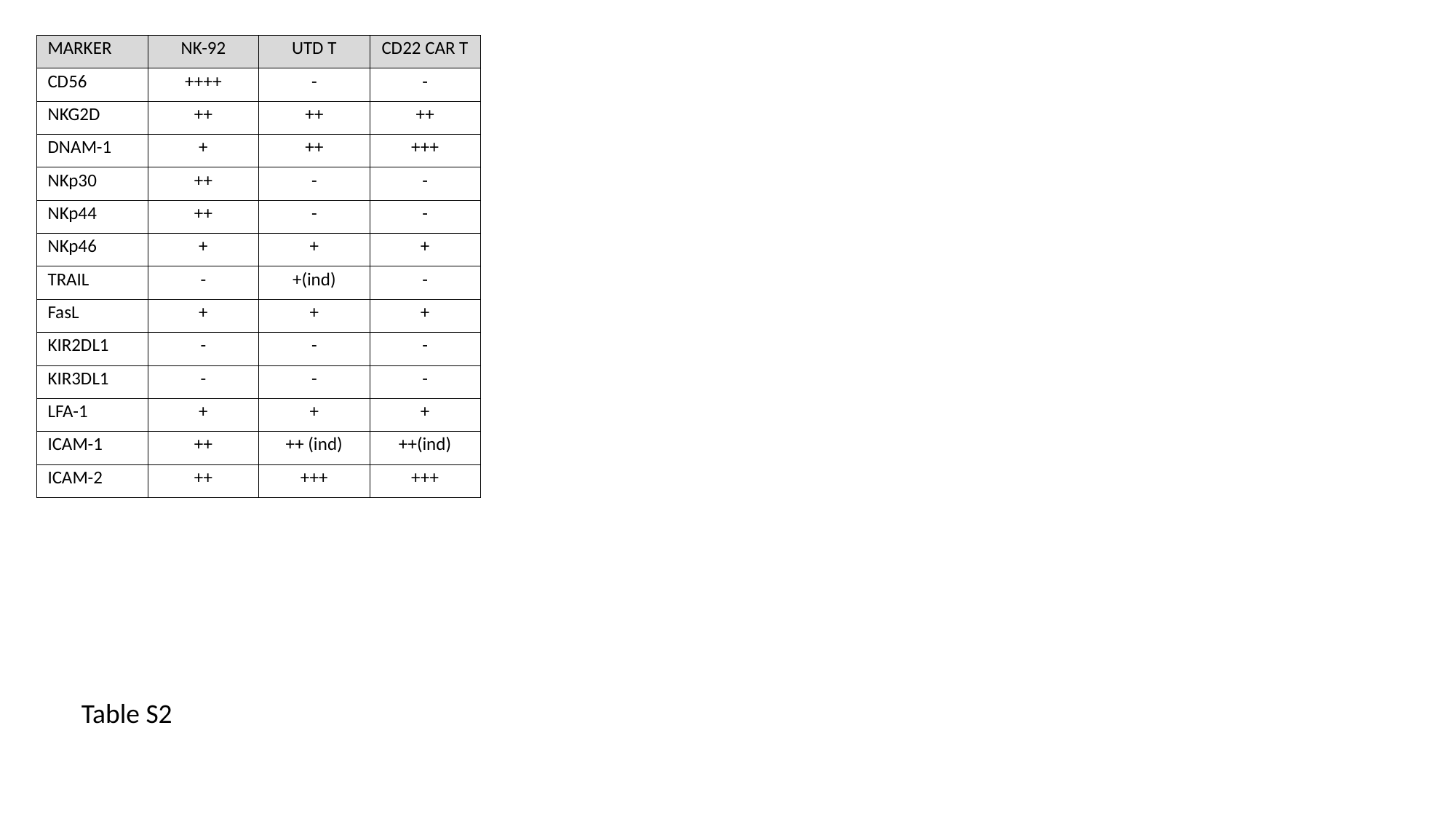

| MARKER | NK-92 | UTD T | CD22 CAR T |
| --- | --- | --- | --- |
| CD56 | ++++ | - | - |
| NKG2D | ++ | ++ | ++ |
| DNAM-1 | + | ++ | +++ |
| NKp30 | ++ | - | - |
| NKp44 | ++ | - | - |
| NKp46 | + | + | + |
| TRAIL | - | +(ind) | - |
| FasL | + | + | + |
| KIR2DL1 | - | - | - |
| KIR3DL1 | - | - | - |
| LFA-1 | + | + | + |
| ICAM-1 | ++ | ++ (ind) | ++(ind) |
| ICAM-2 | ++ | +++ | +++ |
Table S2

## Slide 3
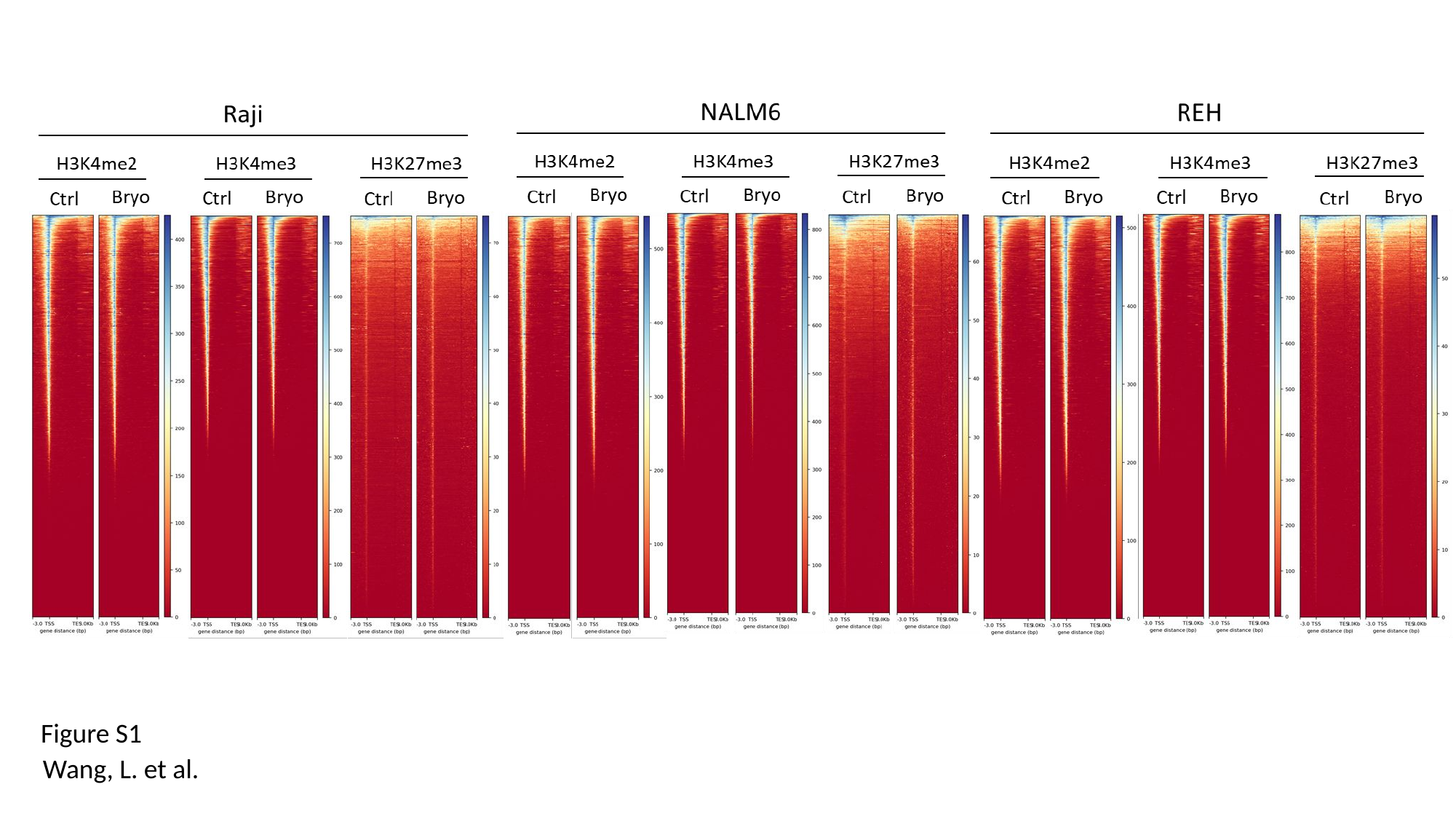

Figure S1
Wang, L. et al.

## Slide 4
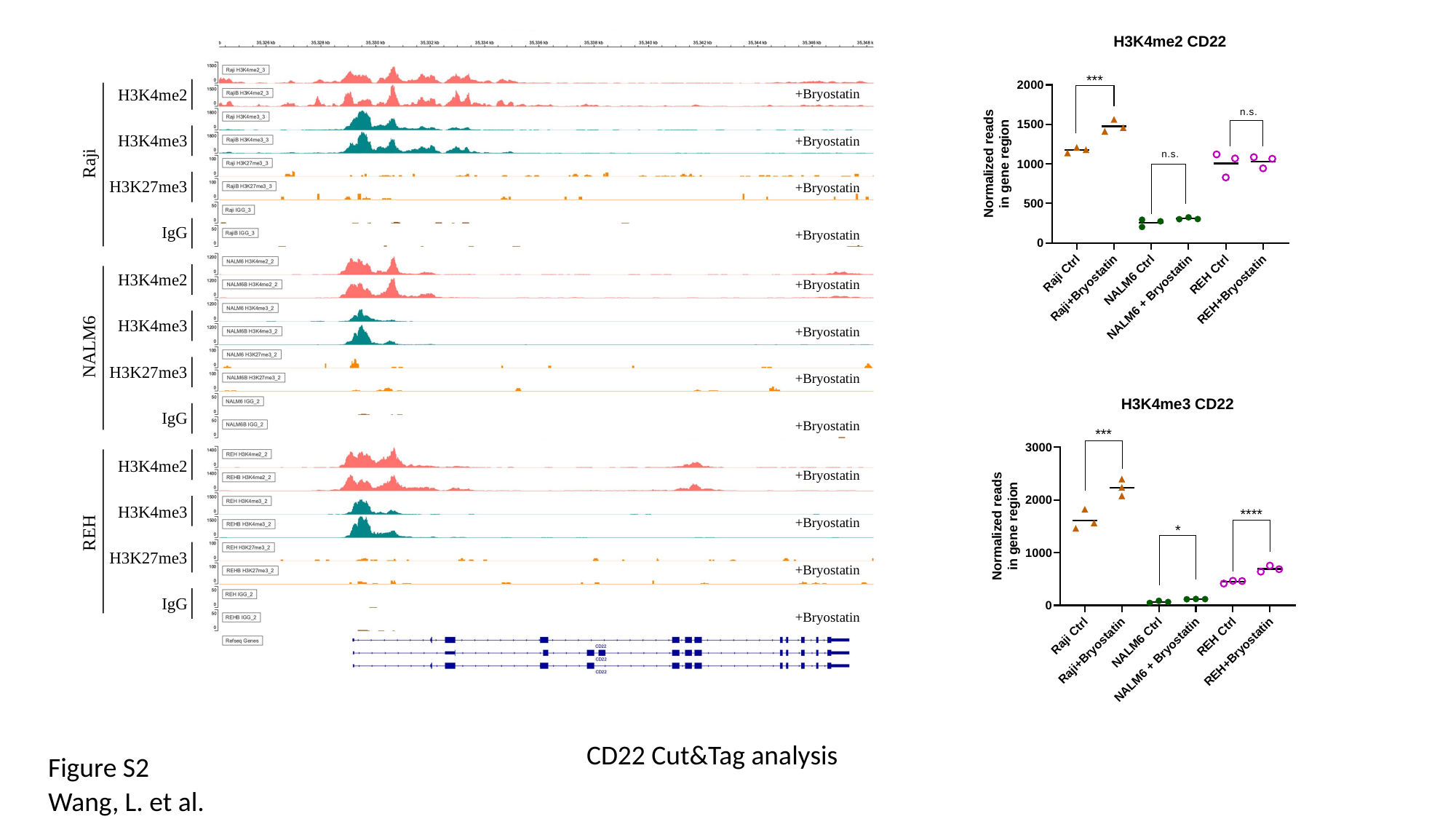

H3K4me2
H3K4me3
Raji
H3K27me3
IgG
H3K4me2
H3K4me3
NALM6
H3K27me3
IgG
H3K4me2
H3K4me3
REH
H3K27me3
IgG
+Bryostatin
+Bryostatin
+Bryostatin
+Bryostatin
+Bryostatin
+Bryostatin
+Bryostatin
+Bryostatin
+Bryostatin
+Bryostatin
+Bryostatin
+Bryostatin
CD22 Cut&Tag analysis
Figure S2
Wang, L. et al.

## Slide 5
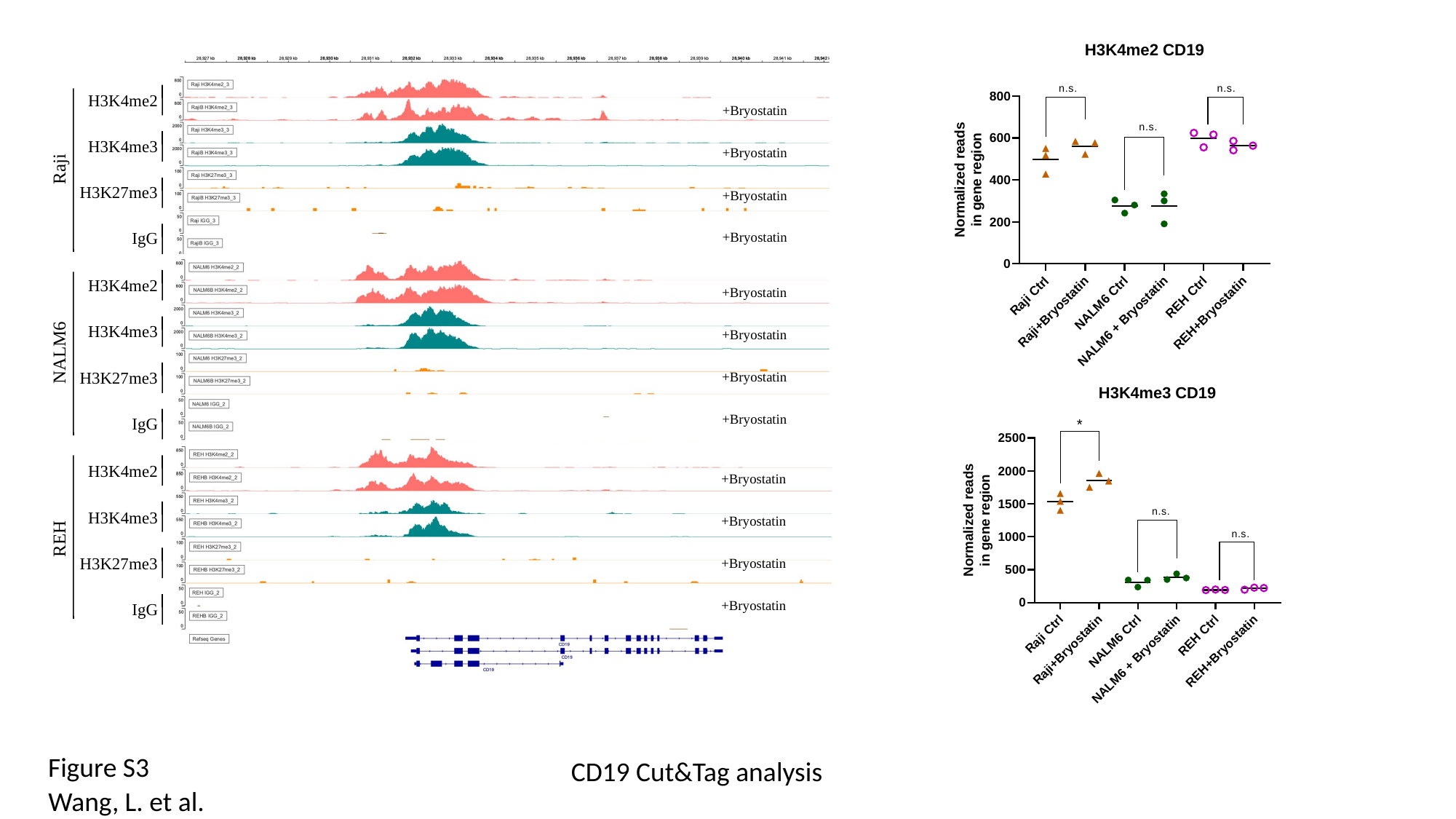

H3K4me2
H3K4me3
Raji
H3K27me3
IgG
H3K4me2
H3K4me3
NALM6
H3K27me3
IgG
H3K4me2
H3K4me3
REH
H3K27me3
IgG
+Bryostatin
+Bryostatin
+Bryostatin
+Bryostatin
+Bryostatin
+Bryostatin
+Bryostatin
+Bryostatin
+Bryostatin
+Bryostatin
+Bryostatin
+Bryostatin
Figure S3
CD19 Cut&Tag analysis
Wang, L. et al.

## Slide 6
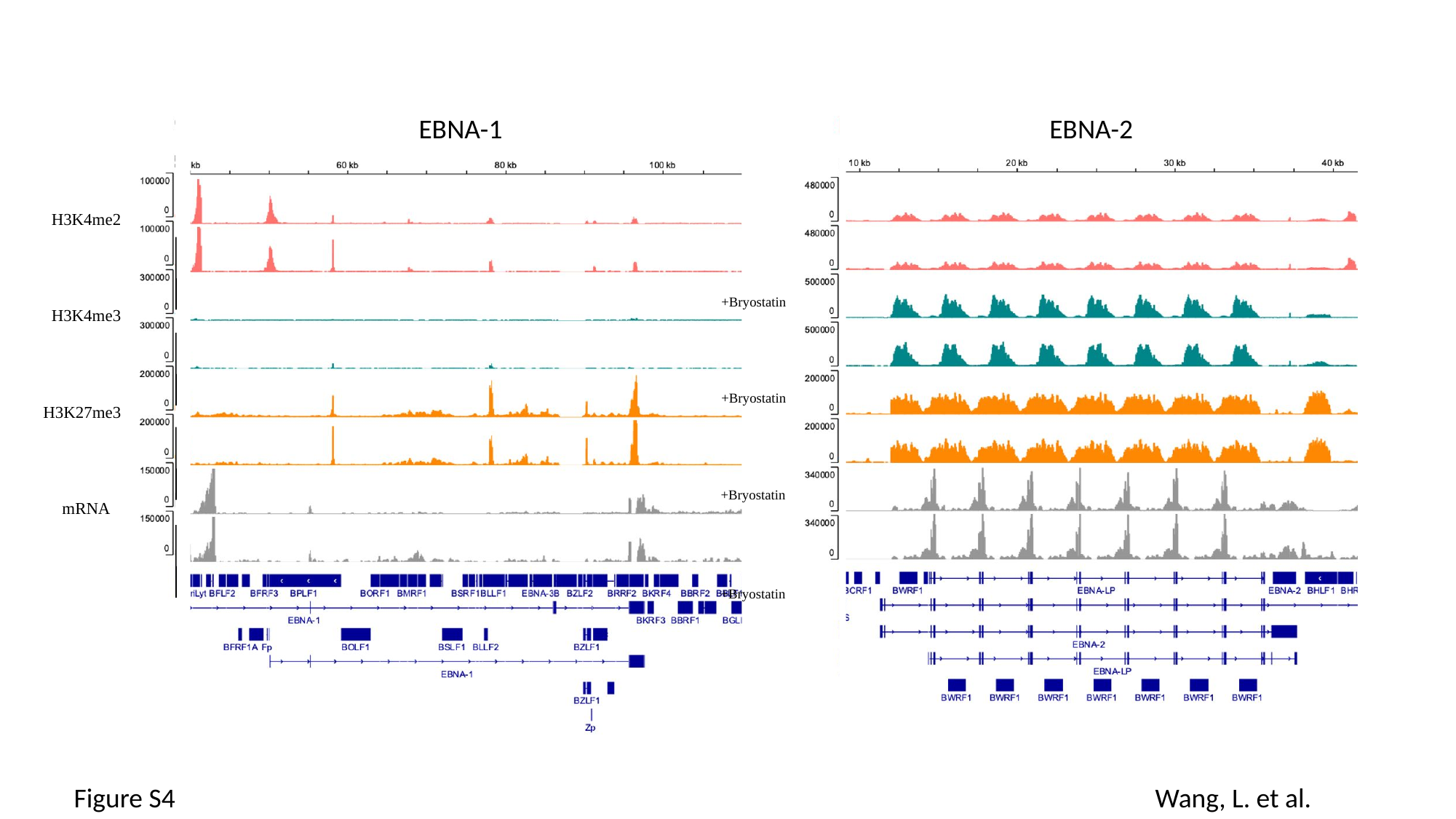

EBNA-1
EBNA-2
H3K4me2
+Bryostatin
H3K4me3
+Bryostatin
H3K27me3
+Bryostatin
mRNA
+Bryostatin
Figure S4
Wang, L. et al.

## Slide 7
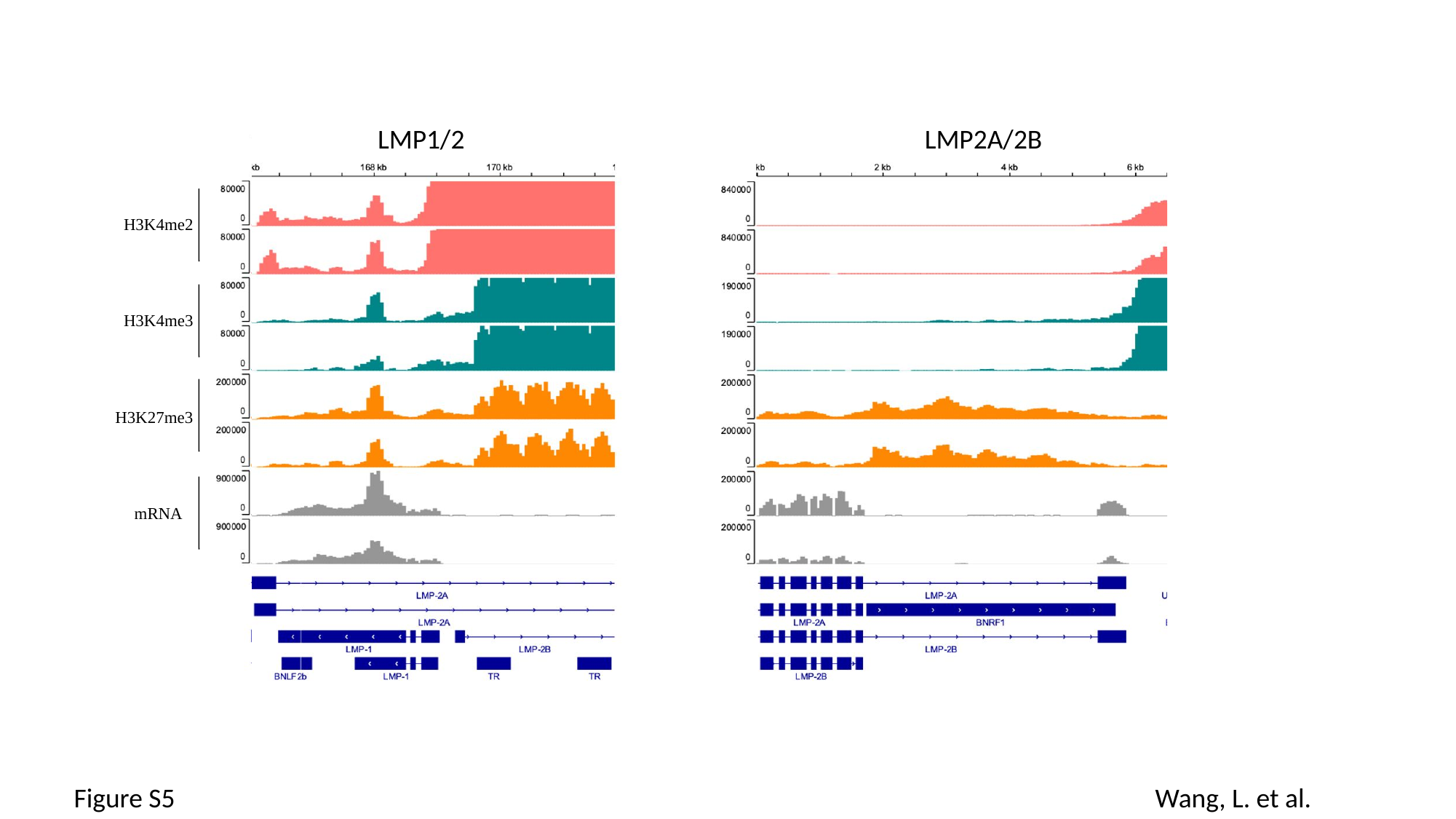

LMP1/2
LMP2A/2B
H3K4me2
H3K4me3
H3K27me3
mRNA
Figure S5
Wang, L. et al.

## Slide 8
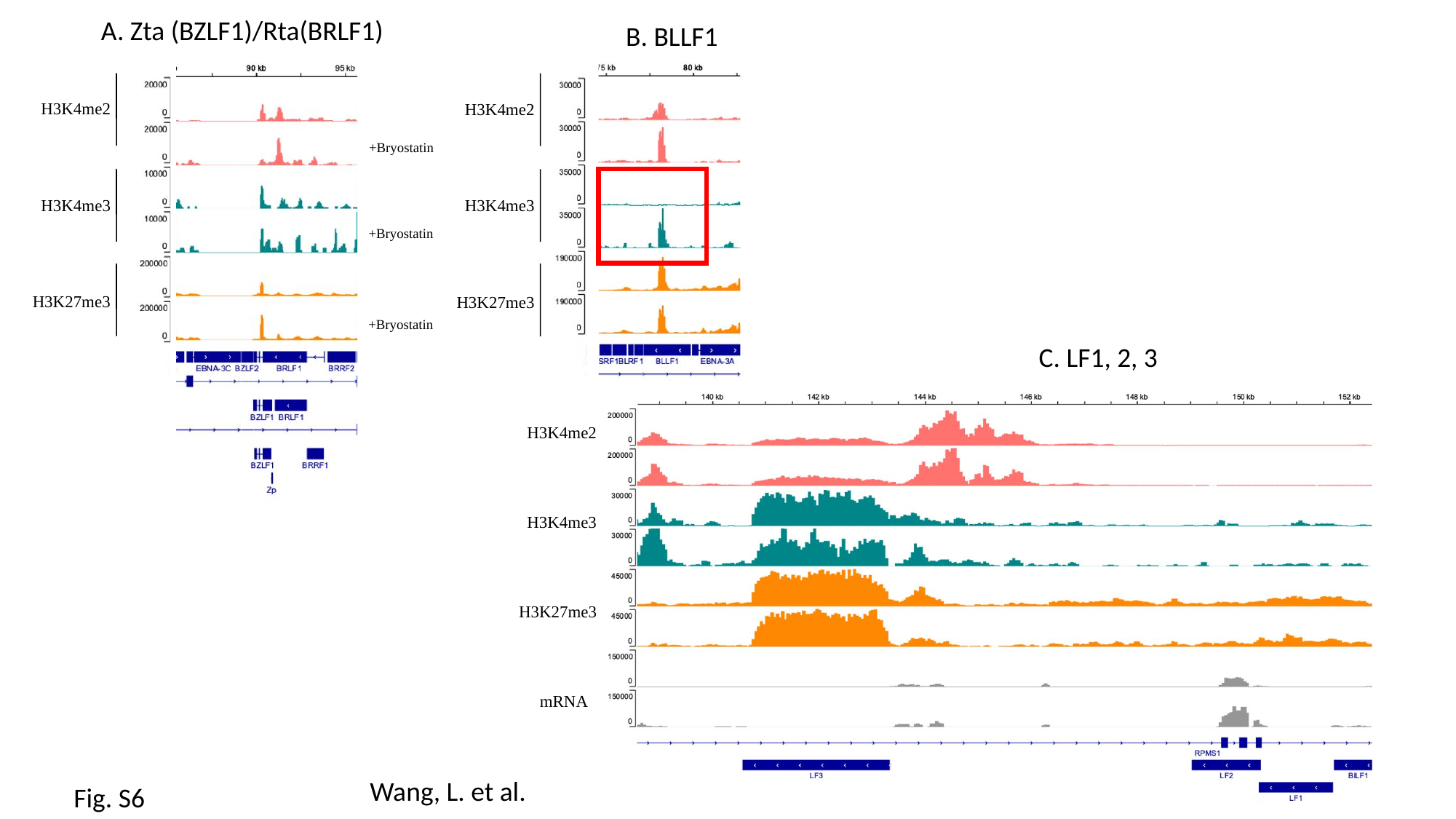

A. Zta (BZLF1)/Rta(BRLF1)
H3K4me2
H3K4me3
+Bryostatin
+Bryostatin
+Bryostatin
H3K27me3
B. BLLF1
H3K4me2
H3K4me3
H3K27me3
C. LF1, 2, 3
H3K4me2
H3K4me3
H3K27me3
mRNA
Wang, L. et al.
Fig. S6

## Slide 9
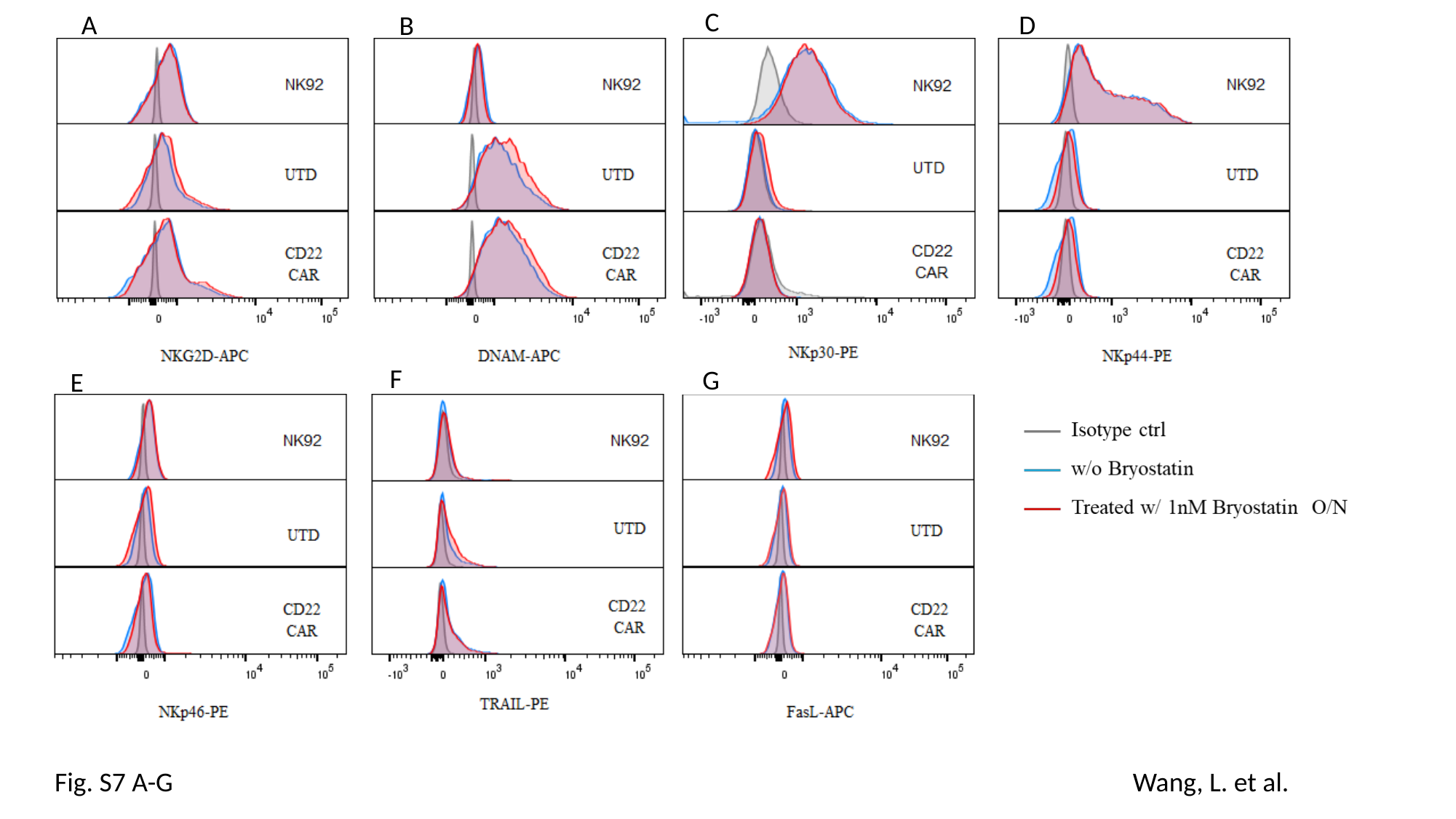

C
A
D
B
F
G
E
Fig. S7 A-G
Wang, L. et al.

## Slide 10
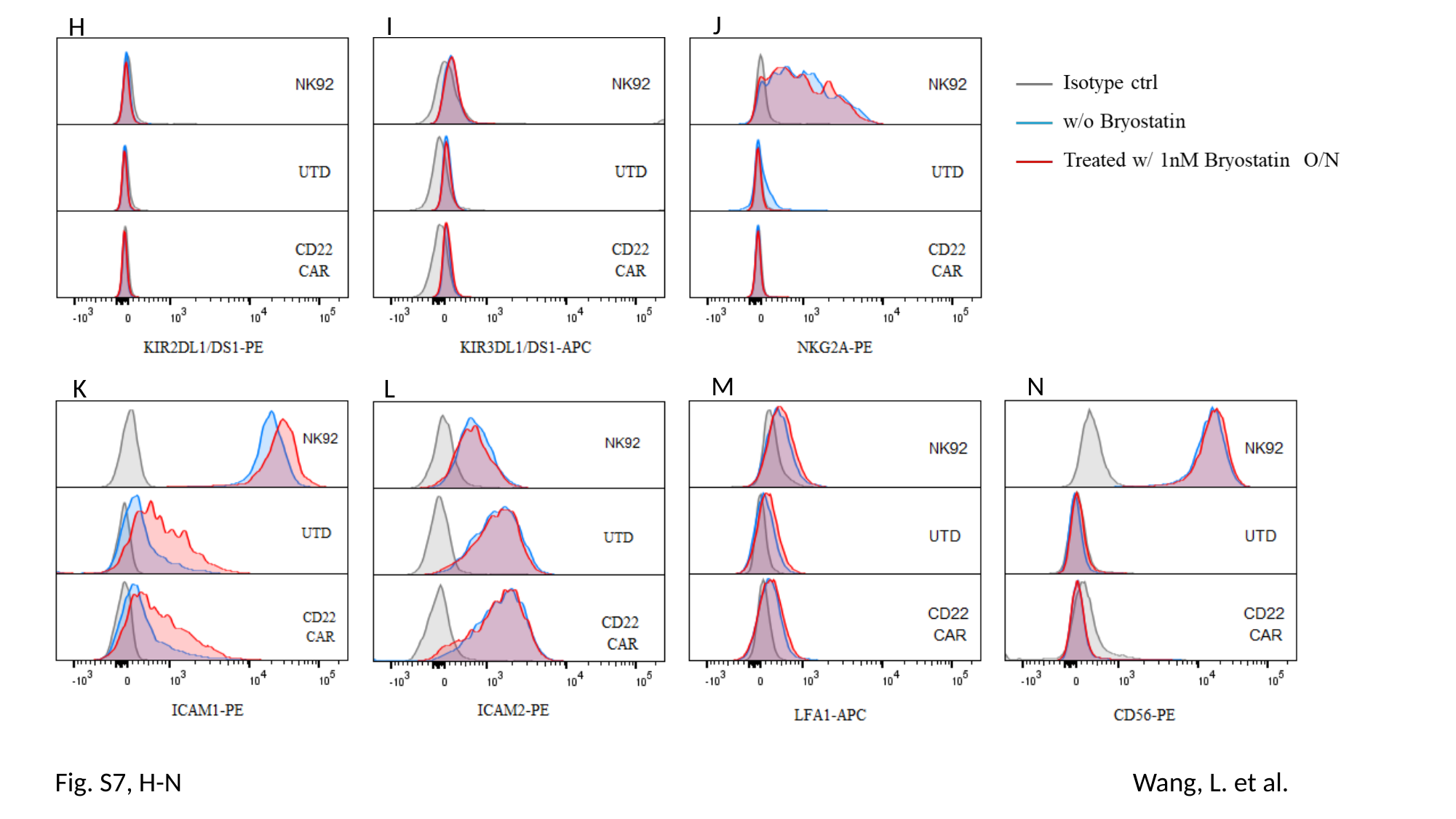

J
I
H
M
N
K
L
Fig. S7, H-N
Wang, L. et al.

## Slide 11
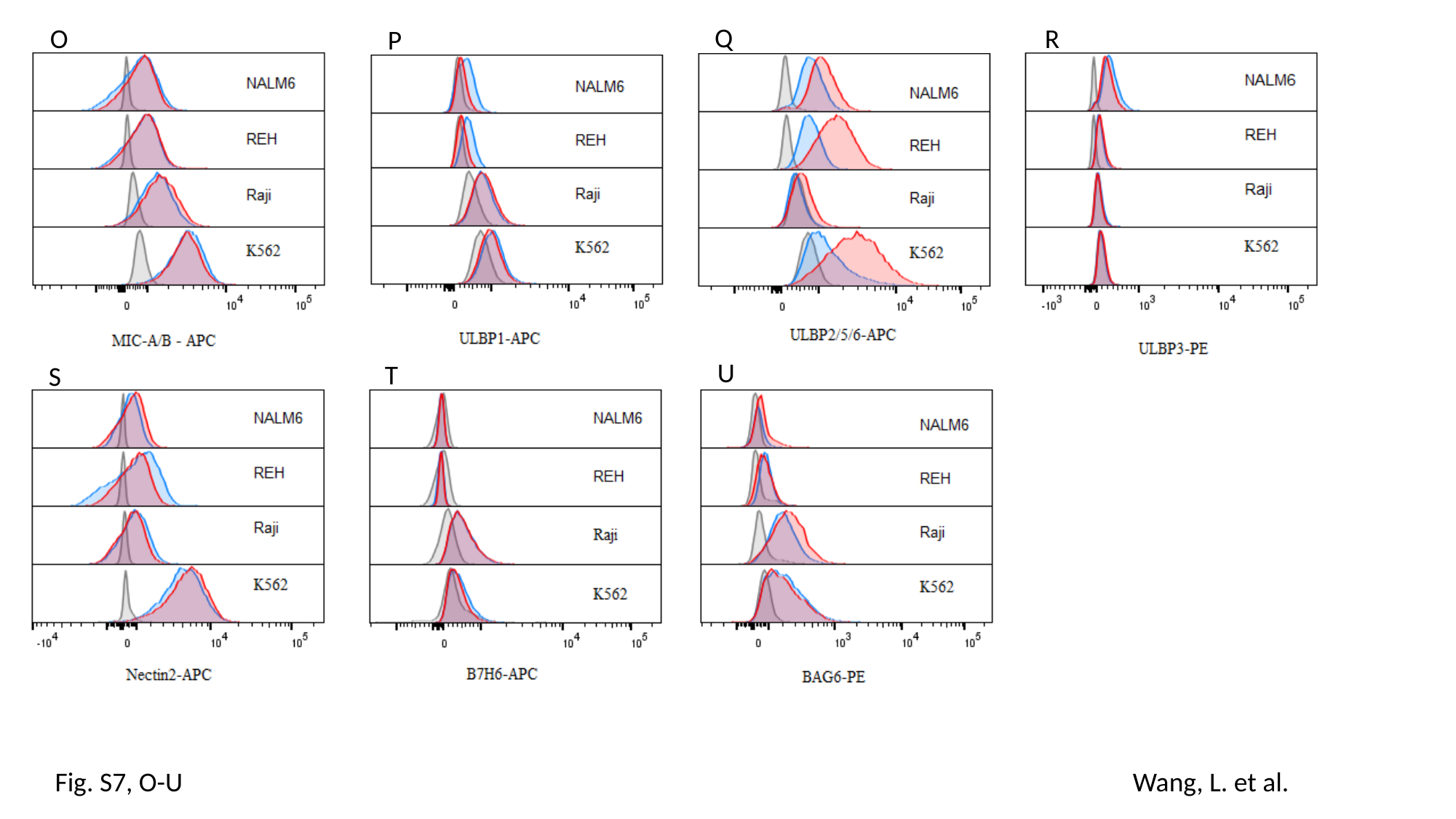

Q
O
R
P
U
T
S
Fig. S7, O-U
Wang, L. et al.

## Slide 12
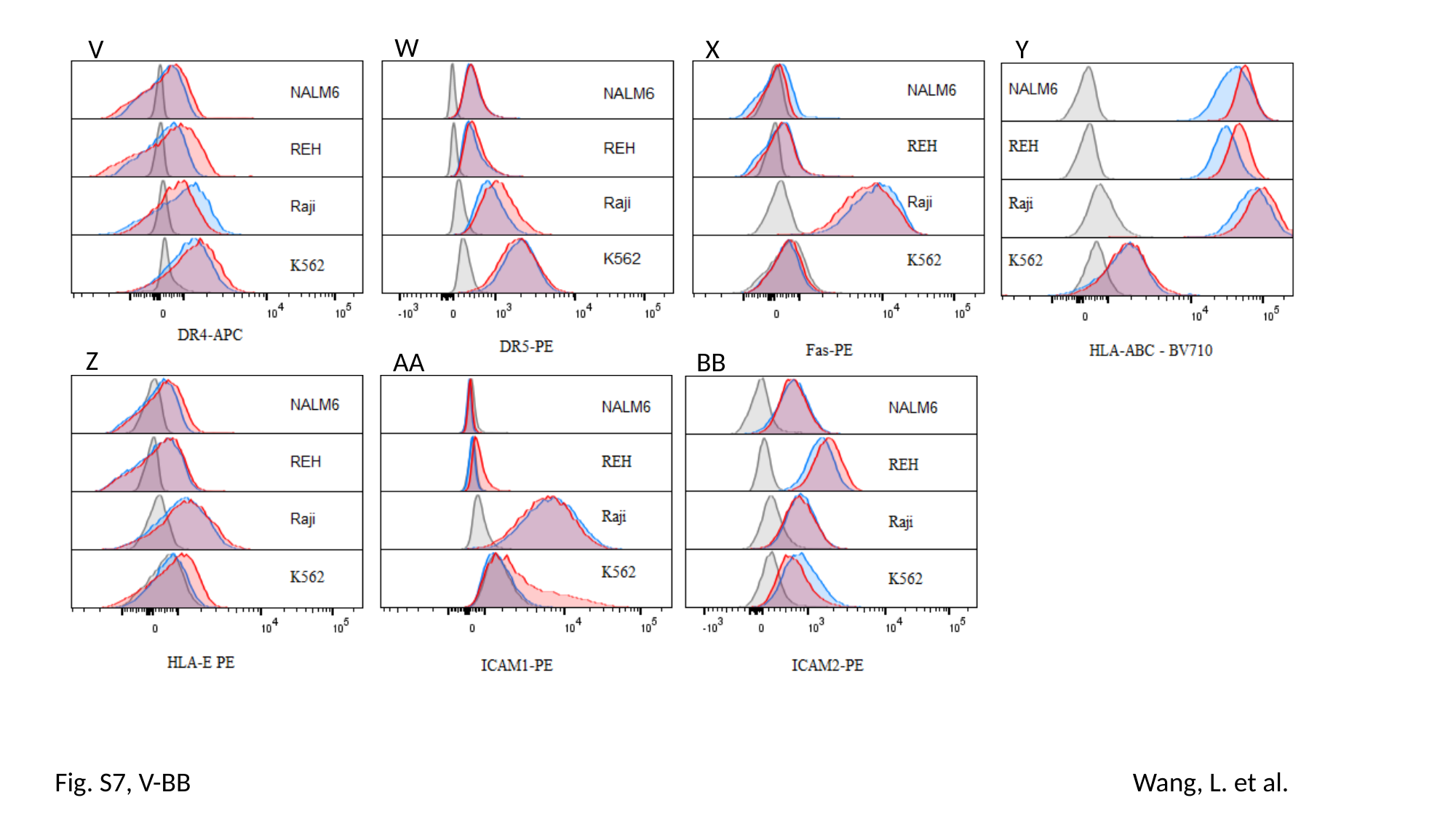

W
V
X
Y
Z
AA
BB
Fig. S7, V-BB
Wang, L. et al.

## Slide 13
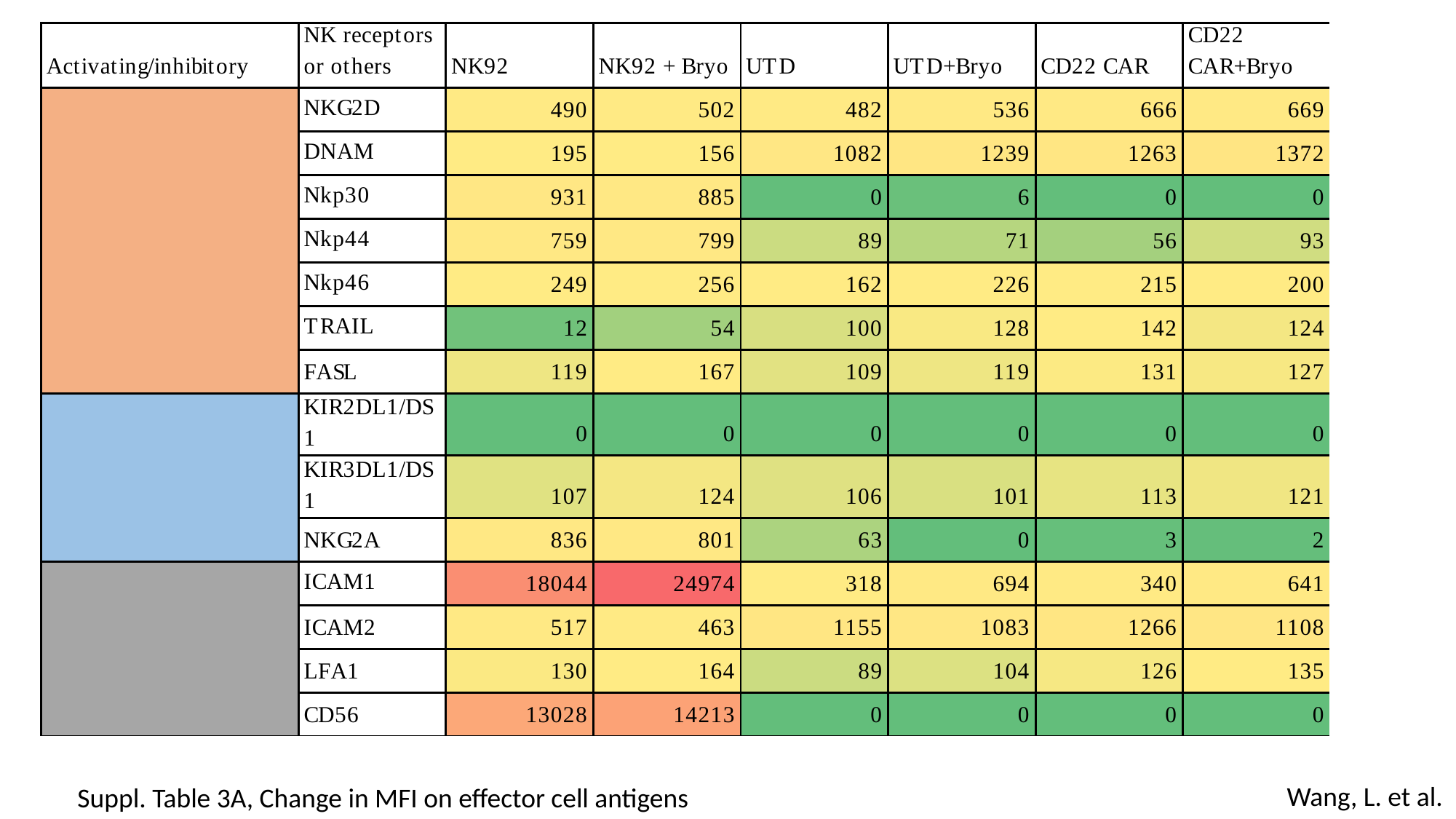

Wang, L. et al.
Suppl. Table 3A, Change in MFI on effector cell antigens

## Slide 14
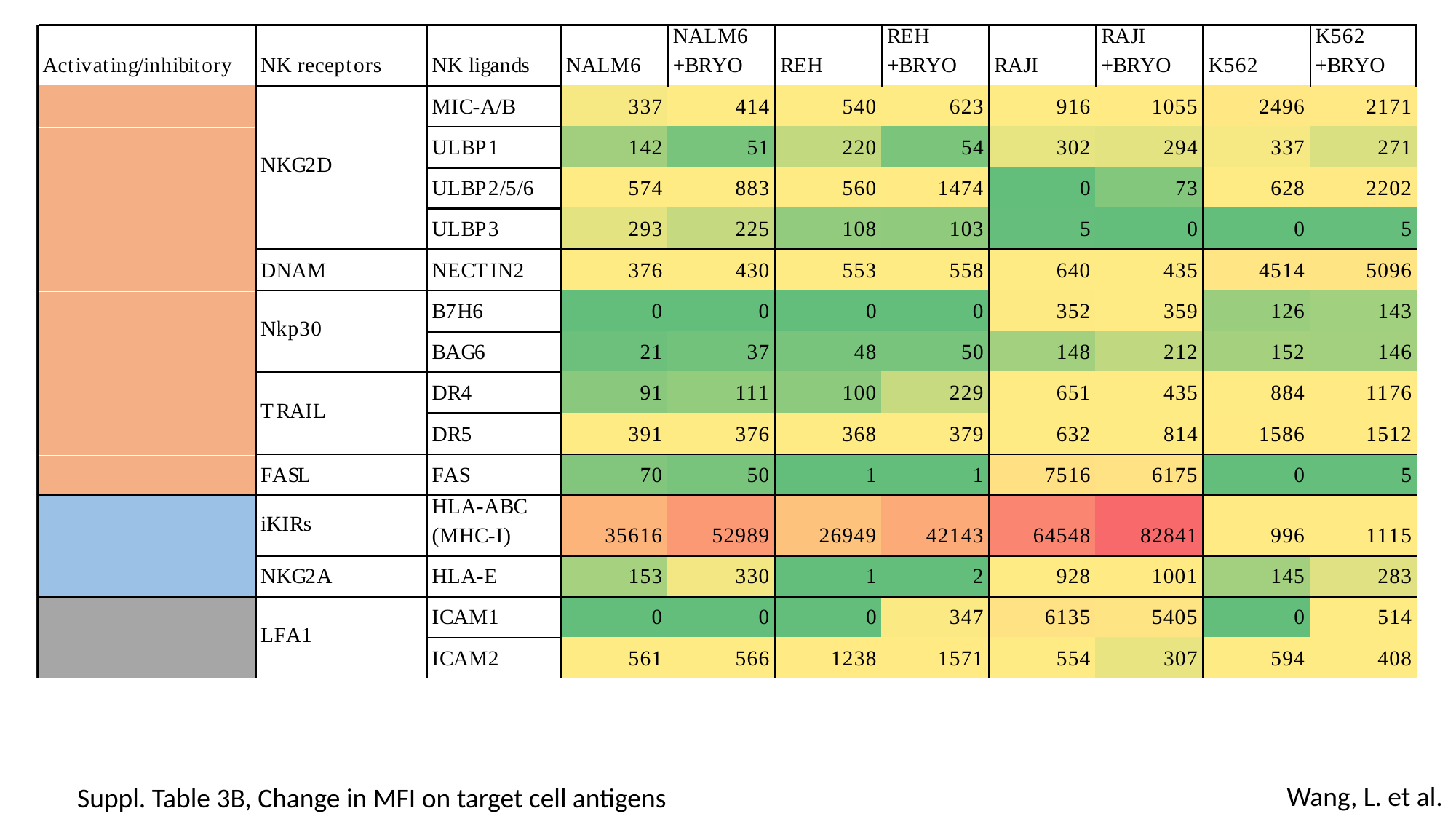

Wang, L. et al.
Suppl. Table 3B, Change in MFI on target cell antigens

## Slide 15
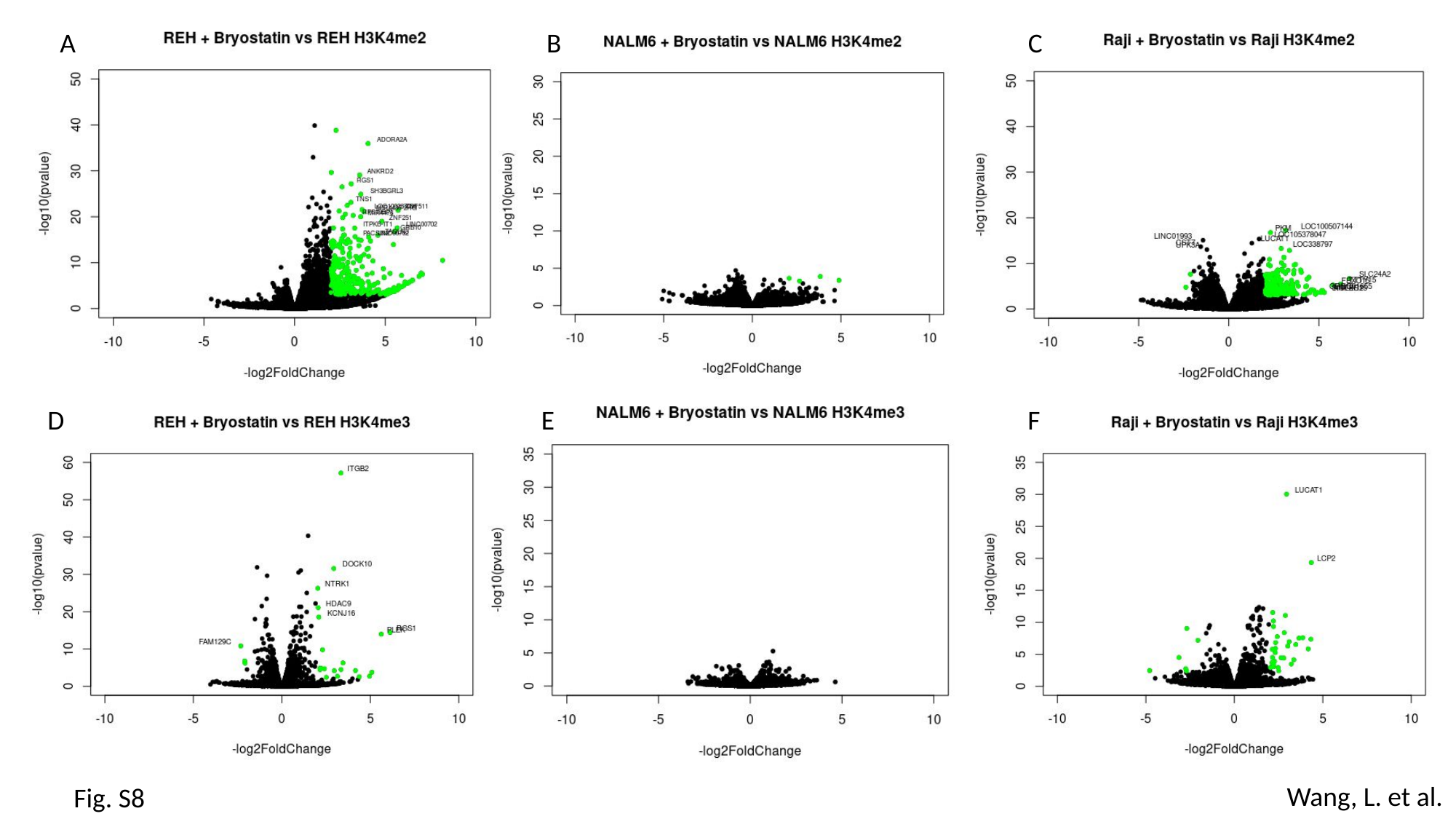

A
B
C
D
E
F
Wang, L. et al.
Fig. S8
